# Design Optimization of Resource Allocation in OFDMA-Based Cognitive Radio-Enabled Internet of Vehicles (IoVs) †

**DOI:** 10.3390/s20216402

**Published:** 2020-11-09

**Authors:** Joy Eze, Sijing Zhang, Enjie Liu, Elias Eze

**Affiliations:** Institute for Research in Applicable Computing (IRAC), School of Computer Science and Technology, University of Bedfordshire, Luton LU1 3JU, UK; sijing.zhang@beds.ac.uk (S.Z.); enjie.liu@beds.ac.uk (E.L.); elias.eze1@beds.ac.uk (E.E.)

**Keywords:** Cognitive Radio, game theory, Internet of Vehicles, OFDMA, vehicular networks

## Abstract

Joint optimal subcarrier and transmit power allocation with QoS guarantee for enhanced packet transmission over Cognitive Radio (CR)-Internet of Vehicles (IoVs) is a challenge. This open issue is considered in this paper. A novel SNBS-based wireless radio resource scheduling scheme in OFDMA CR-IoV network systems is proposed. This novel scheduler is termed the SNBS OFDMA-based overlay CR-Assisted Vehicular NETwork (SNO-CRAVNET) scheduling scheme. It is proposed for efficient joint transmit power and subcarrier allocation for dynamic spectral resource access in cellular OFDMA-based overlay CRAVNs in clusters. The objectives of the optimization model applied in this study include (1) maximization of the overall system throughput of the CR-IoV system, (2) avoiding harmful interference of transmissions of the shared channels’ licensed owners (or primary users (PUs)), (3) guaranteeing the proportional fairness and minimum data-rate requirement of each CR vehicular secondary user (CRV-SU), and (4) ensuring efficient transmit power allocation amongst CRV-SUs. Furthermore, a novel approach which uses Lambert-W function characteristics is introduced. Closed-form analytical solutions were obtained by applying time-sharing variable transformation. Finally, a low-complexity algorithm was developed. This algorithm overcame the iterative processes associated with searching for the optimal solution numerically through iterative programming methods. Theoretical analysis and simulation results demonstrated that, under similar conditions, the proposed solutions outperformed the reference scheduler schemes. In comparison to other scheduling schemes that are fairness-considerate, the SNO-CRAVNET scheme achieved a significantly higher overall average throughput gain. Similarly, the proposed time-sharing SNO-CRAVNET allocation based on the reformulated convex optimization problem is shown to be capable of achieving up to 99.987% for the average of the total theoretical capacity.

## 1. Introduction

In the near future, most vehicles are expected to be equipped with wireless communication technologies, such as On-Board Units (OBUs), and ultrasonic sensors to enable a variety of new services, such as safety applications, improved traffic management, and enhanced infotainment services [1]. Therefore, the vehicular ad hoc network (VANET) has gained an increased importance, receiving a great amount of attention from academia, auto-manufacturing industries, and government agencies. Wireless Access in Vehicular Environments (WAVEs) is a recently approved protocol suite for wireless communication in vehicular networks, and it relies on IEEE 802.11p at the medium access control (MAC) and physical (PHY) layers. Accordingly, the IEEE 1609.4 protocol stack has been approved by an IEEE delegated Working Group (WG), in order to provide an efficient mechanism for multi-channel operations in WAVEs, where the control channel (CCH) and service channels (SCHs) are periodically synchronized at intervals [2].

All vehicles are expected to contend for channel access over the 75 MHz spectrum allocated by the US Federal Communication Commission (FCC) in the 5.9 GHz spectrum band for the WAVE system and use it for the exchange of safety and infotainment information. However, to realize the full potential of Internet of Vehicles (IoVs), intelligent vehicles must be able to wirelessly exchange communication with one another via vehicle-to-vehicle (V2V), vehicle-to-roadside infrastructure (V2I), and vehicle-to-pedestrian handheld device (V2X) communications [2]. They can do this by taking advantage of the wide range of wireless networks and spectra, such as cellular and Wi-Fi networks, TV bands, and satellite networks, depending on their availability and the location of intelligent vehicles. On the contrary, the anticipated increase in demand for diverse vehicular network-oriented applications (safety- and non-safety-related services) would certainly result in a shortage of spectral resources for IoV communication networks.

According to [2,3], the emerging Cognitive Radio (CR) technology has been envisaged as an enabling concept with the potential to overcome the challenge of spectrum scarcity, which is the result of the existing fashion of fixed spectrum allocation (FSA) policy [4]. Dynamic spectrum access (DSA) or the spectrum sharing mechanism [5] has been adjudged a vital potential associated with CR technology. Additionally, the existing allocation of the spectrum for certain radio technologies within 300 MHz–3 GHz (i.e., the prime frequency bands) is getting closer to the saturation point. Consequently, spectrum allocation regulatory bodies such as the UK Office of Communications (Ofcom) or the US FCC are considering more flexible spectrum management strategies, such as the secondary spectrum access mechanism [2]. Therefore, the design and development of unique novel radio technologies such as DSA or the spectrum sharing mechanism [5,6] are vital, in order to be able to conduct operations in unlicensed bands.

Game theory is seen as a robust tool for achieving Pareto-optimality for distributed resource scheduling, especially within wireless networks such as vehicular networks [7,8], as well as within the CR network [9,10,11,12]. Furthermore, when efficient spectrum resource sharing is considered in conjunction with fairness, it has been figured out that a cooperative game theoretical technique such as the Nash bargaining solution (NBS) is more suitable for finding the optimal points in comparison to non-cooperative games.

This paper’s emphasis is on the efficient allocation of spectrum resources with the assumption that the intelligent detection of spectrum holes [2,13] is carried out in advance. The key aim is to efficiently schedule the dynamically available spectrum resources with the satisfaction of QoS requirements of the CR vehicular secondary users (CRV-SUs), while guaranteeing non-interference of potential communications from the licensed primary users (PUs). Using DSA, CRV-SUs can intelligently detect the presence of spectral white spaces (i.e., available spectrum holes) that are temporarily left under-utilized by the licensed owner (i.e., the PUs), dynamically utilize the available spectrum, and vacate in the instance of the licensed PUs. In this study, a cellular OFDMA-based overlay CR-Assisted Vehicular NETwork scenario is considered, where licensed spectrum owners permit CRV-SUs to access the unused spectral resources, providing the licensed PUs with a guarantee of no performance degradation. Therefore, in an overlay fashion, CRV-SUs are not permitted to access the sub-channels that are under current PU occupation and this ensures that there is no co-channel interference between the PUs and CRV-SUs.

It is possible for both PUs and CRV-SUs to exist in side by side frequency bands in an overlay fashion, possibly using different access technologies. Therefore, the signals transmitted by both PUs and CRV-SUs are not orthogonal. Considering this, the non-orthogonality of the signals may lead to mutual interference amongst the PUs and CRV-SUs. Consequently, the measure of the introduced interference of the PUs by the CRV-SUs is directly proportional to the amount of transmit power allocation in the CRV-SUs’ sub-channels and the difference in the spectral distance that exists between the PU’s band and the sub-channels. Therefore, spectral resource allocation under cellular OFDMA-based overlay CR-Assisted Vehicular NETworks promises an efficient approach that is capable of protecting the licensed PUs from harmful interference from the CRV-SUs and the potential to satisfy the QoS requirements of CRV-SUs, especially for the communication of time-constrained safety (or emergency) messages in IoVs. The choice of cellular OFDMA-based overlay CR-Assisted Vehicular NETworks is in accordance with the current trends of developments with respect to future wireless communication systems, such as IEEE 802.16-style networks, IEEE 802.11p networks, and universal mobile telecommunication system (UMTS) long-term evolution (LTE). Moreover, multicarrier OFDMA technology can lead to improvements of the spectral efficiency, as well as the robustness needed when dealing with time-varying wireless multi-path interferences, which is likely the case with vehicular networks [14]. Table 1 and Table 2 present the key mathematical notations and acronyms used in this study and their meaning, respectively.

In this paper, a novel Symmetric NBS OFDMA-based overlay CR-Assisted Vehicular NETwork (SNO-CRAVNET) scheduling scheme is proposed for efficient joint transmit power and subcarrier allocation for dynamic spectral resource access in cellular OFDMA-based overlay CR-Assisted Vehicular NETworks in clusters. The joint optimal allocation strategy is determined in a simpler and faster approach by the proposed scheduler, with the help of the obtained closed-form analytical solution, as opposed to previous studies, which have adopted iterative programming methods, such as the work presented in [15,16,17,18]. Under the interweave-based CR-enabled IoV network systems, the spectrum sensing accuracy remains an open issue due to prevailing sensing errors over wireless channels. However, the scope of this study does not cover an investigation of the integration of spectrum sensing in interweave-based CR-enabled IoV network systems. The merits of the proposed novel SNO-CRAVNET scheme are confirmed through its comparison with existing approaches.

## 2. System Model

**Figure 1:**
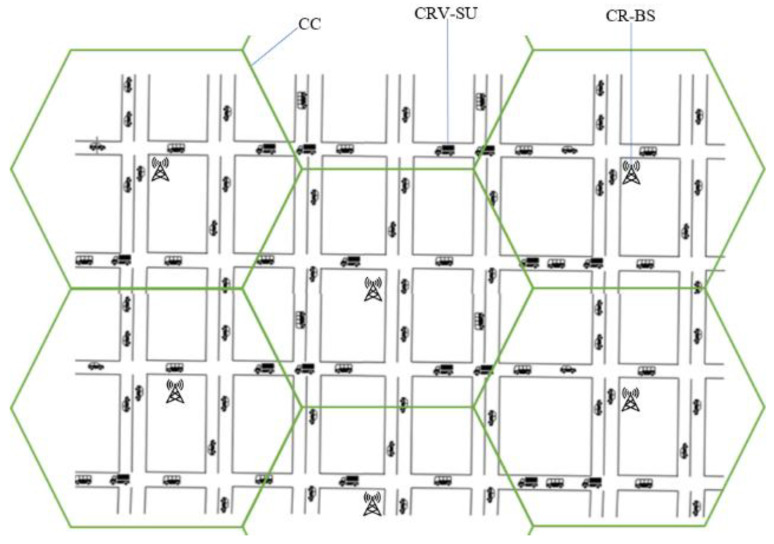
Illustration of a typical cognitive cell (CC) service area.

**Figure 2:**
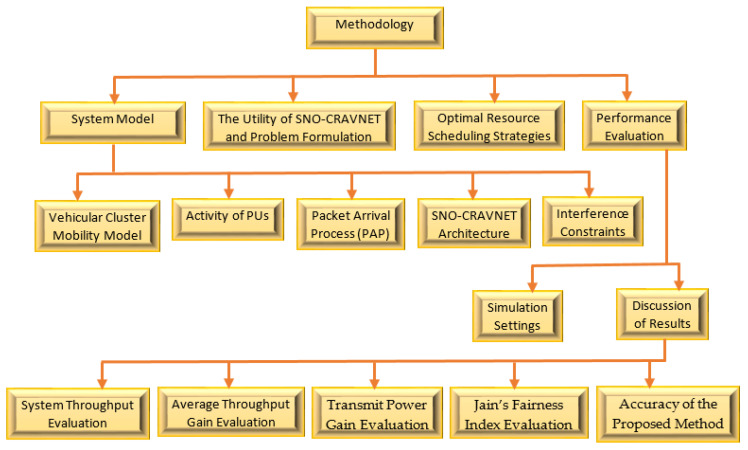
Illustration of the phases of the research study.

The co-existence of the cellular OFDMA-based overlay CR-Assisted Vehicular NETwork with the PU network scenario as depicted in Figure 1 is considered in this paper. As demonstrated in Figure 1, the network scenario is divided into cognitive cells (CCs) [2]. The CCs consist of ℕ number of CR base stations (CR-BS) and ℝ number of CRV-SUs in vehicular cluster formations. The dynamically available channel bandwidth (B) is evenly divided within a given CC into $N_{C}$ number of orthogonal channels. Specifically, the CR-BS in-charge of a CC receives data from CRV-SUs and efficiently performs spectral resource scheduling. Therefore, to prevent harmful interference with the transmissions of the PUs, the CR-BS controls the dynamically available resources. Figure 2 presents an illustration of the phases of the research carried out in this study. The study is divided into four main phases: The system model; proposed utility of SNO-CRAVNET and problem formulation; optimal resource scheduling strategies; and performance evaluation. The system model is further sub-divided into sub-phases, such as the vehicular cluster mobility model, activity of PUs, packet arrival process (PAP), SNO-CRAVNET architecture, and interference constraints. Each of these research phases and sub-phases are detailed in the following sections and sub-sections.

### 2.1. Vehicular Cluster Mobility Model

The average life span of CRV-SUs’ cluster formation [1] is the total duration when all the ℝ CRV-SUs in a particular cluster (usually presumed to be exponentially distributed) maintain membership of the same CC [19]. Therefore, the CRV-SUs’ cluster formation mobility can be modeled using a transition rate (i.e., speed) matrix Ɱ and given by Equation (1)

(1) $$Ɱ=\left[\begin{array}{ccc} ɱ\left(1,\;1\right) & \cdots & ɱ\left(1,j_{m}\right)\\ \vdots & \ddots & \vdots \\ ɱ\left(j_{m},\;1\right) & \cdots & ɱ\left(j_{m},j_{m}\right) \end{array}\right]$$

where $j_{m}=\left|J\right|$ denotes the number of locations in a service area, |J| represents the cardinality of set J, and the element $ɱ\left(j,\;j^{\prime }\right)$ represents the rate at which the CRV-SUs’ cluster formation changes from $L_{j}$ to $L_{j^{\prime }}$ location. Different speeds of CRV-SUs’ cluster formation can be captured in different locations by the matrix Ɱ within a service area.

We represent the steady state probability vector with $\vec{Ṿ}={\left[M\left(L_{1}\right)\cdots M\left(L_{j}\right)\cdots M\left(L_{j_{m}}\right)\right]}^{t}$, where the element M(L) of the vector $\vec{Ṿ}$ denotes the probability that the CRV-SUs’ cluster formation occurs at the location $L_{j}$. Therefore, by solving $\vec{Ṿ}{}^{t}Ɱ=\vec{0}$ and $\vec{Ṿ}{}^{t}\vec{1}=1$, the steady state probability vector $\vec{Ṿ}$ can be obtained, where $\vec{1}$ and $\vec{0}$ represent the vectors of ones and zeros, respectively.

### 2.2. Activity of PUs

Packet transmission from both CRV-SUs’ cluster members (CMs) to cluster heads (CHs) and from the CHs to their respective destinations is based on the common channel shared with the licensed users. Therefore, both the CRV-SUs’ CMs and the CHs must always watch the activity of the licensed users prior to accessing and using the shared channel in a fashion that does not cause harmful interference of the PUs’ activity. In a shared channel, the activity of the PUs is modeled through a two-state Markov chain (see Equation (2)), such as the ON-OFF model, which corresponds to the *busy* and *idle* states, respectively. Consequently, a transition probability matrix is used to model the state transition of shared channel m, as expressed below:

(2) $${\tilde{T}}_{m}=\left[\begin{array}{cc} {\tilde{T}}_{m}\left(0,\;0\right) & {\tilde{T}}_{m}\left(0,\;1\right)\\ {\tilde{T}}_{m}\left(1,\;0\right) & {\tilde{T}}_{m}\left(1,\;1\right) \end{array}\right]\begin{array}{c} \leftarrow \mathrm{idle}\\ \leftarrow \mathrm{busy} \end{array}\;$$

where 0 and 1 represent the idle and busy states, respectively. Therefore, the probability of the shared wireless channel m being in an idle state $P_{m}^{idle}$ can be obtained from the expression $P_{m}^{idle}=1-{\tilde{T}}_{m}\left(1,\;1\right)/\left(\left({\tilde{T}}_{m}\left(0,\;1\right)-{\tilde{T}}_{m}\left(1,\;1\right)+1\right)\right)$.

Conversely, the sensed state (i.e., idle or busy state) of the shared wireless channel m may differ from the actual channel state because of shared channel sensing error. Therefore, the probability of misdetection for the shared channel sensing, which is the probability that a shared wireless channel m is sensed as idle when it is actually busy, is represented by $P_{m}^{mis}$, and the probability of false-alarm, which is the probability that a shared wireless channel m is sensed as busy when it is actually idle, is represented by $P_{m}^{false}$ [2]. Therefore, considering the inter-relation between the sensed and actual shared wireless channel m states, the transition of the joint sensed and actual shared wireless channel m state can be given as a matrix (see Equation (3)).

(3) $${\tilde{T}}_{m}=\left[\begin{array}{cccc} {\tilde{T}}_{m}\left(0,0\right)\left(1-P_{m}^{false}\right) & {\tilde{T}}_{m}\left(0,1\right)P_{m}^{mis} & {\tilde{T}}_{m}\left(0,0\right)P_{m}^{false} & {\tilde{T}}_{m}\left(0,1\right)\left(1-P_{m}^{mis}\right)\\ {\tilde{T}}_{m}\left(1,0\right)\left(1-P_{m}^{false}\right) & {\tilde{T}}_{m}\left(1,1\right)P_{m}^{mis} & {\tilde{T}}_{m}\left(1,0\right)P_{m}^{false} & {\tilde{T}}_{m}\left(1,1\right)\left(1-P_{m}^{mis}\right)\\ {\tilde{T}}_{m}\left(0,0\right)\left(1-P_{m}^{false}\right) & {\tilde{T}}_{m}\left(0,1\right)P_{m}^{mis} & {\tilde{T}}_{m}\left(0,0\right)P_{m}^{false} & {\tilde{T}}_{m}\left(0,1\right)\left(1-P_{m}^{mis}\right)\\ {\tilde{T}}_{m}\left(1,0\right)\left(1-P_{m}^{false}\right) & {\tilde{T}}_{m}\left(1,1\right)P_{m}^{mis} & {\tilde{T}}_{m}\left(0,0\right)P_{m}^{false} & {\tilde{T}}_{m}\left(1,1\right)\left(1-P_{m}^{mis}\right) \end{array}\right]\begin{array}{c} \leftarrow_{\;\mathrm{and}\;\mathrm{sensed}\;idle}^{\mathrm{shared}\;\mathrm{channel}\;idle,}\\ \leftarrow_{\mathrm{but}\;\mathrm{sensed}\;idle}^{\mathrm{shared}\;\mathrm{channel}\;busy,\;}\\ \leftarrow_{\mathrm{but}\;\mathrm{sensed}\;busy}^{\mathrm{shared}\;\mathrm{channel}\;idle,\;}\\ \leftarrow_{\mathrm{and}\;\mathrm{sensed}\;busy}^{\mathrm{shared}\;\mathrm{channel}\;busy,\;} \end{array}$$

Let us assume that the steady state probability vector of the sensed and actual shared wireless channel m state is denoted by $\vec{\delta }$. Therefore, the element δ(i) for i={1, 2, 3, 4} of $\vec{\delta }$ corresponds to the joint sensed and actual shared wireless channel m state as defined in row i of ${\tilde{T}}_{m}$ in Equation (3) above. The steady state probability vector $\vec{\delta }$ can be obtained by solving $\vec{\delta }{}^{t}\left(\vec{1}\right)=1$ and $\vec{\delta }{}^{t}\left({\hat{T}}_{m}\right)=\vec{\delta }{}^{t}$. Considering this, the transition of the sensed shared wireless channel m state can be modeled by the matrix

(4) $$T_{m}=\left[\begin{array}{cc} T_{m}\left(0,0\right) & T_{m}\left(0,1\right)\\ T_{m}\left(1,0\right) & T_{m}\left(1,1\right) \end{array}\right]\begin{array}{c} \leftarrow \mathrm{shared}\;\mathrm{channel}\;\mathrm{sensed}\;idle\\ \leftarrow \mathrm{shared}\;\mathrm{channel}\;\mathrm{sensed}\;busy, \end{array}$$

such that the elements can be obtained as follows (see Equations (5)–(8)):

(5) $$T_{m}\left(0,0\right)=\frac{\left\{\delta \left(1\right)\left({\tilde{T}}_{m}\left(0,0\right)\left(1-P_{m}^{false}\right)+{\tilde{T}}_{m}\left(0,1\right)P_{m}^{mis}\right)+\delta \left(2\right)\left({\tilde{T}}_{m}\left(1,0\right)\left(1-P_{m}^{false}\right)+{\tilde{T}}_{m}\left(1,1\right)P_{m}^{mis}\right)\right\}}{\left\{\delta \left(1\right)+\delta \left(2\right)\right\}}$$

(6) $$T_{m}\left(0,1\right)=\frac{\left\{\delta \left(1\right)\left({\tilde{T}}_{m}\left(0,0\right)P_{m}^{false}+{\tilde{T}}_{m}\left(0,1\right)\left(1-P_{m}^{mis}\right)\right)+\delta \left(2\right)\left({\tilde{T}}_{m}\left(1,0\right)P_{m}^{false}+{\tilde{T}}_{m}\left(1,1\right)\left(1-P_{m}^{mis}\right)\right)\right\}}{\left\{\delta \left(1\right)+\delta \left(2\right)\right\}}$$

(7) $$T_{m}\left(1,0\right)=\frac{\left\{\delta \left(3\right)\left({\tilde{T}}_{m}\left(0,0\right)\left(1-P_{m}^{false}\right)+{\tilde{T}}_{m}\left(0,1\right)P_{m}^{mis}\right)+\delta \left(4\right)\left({\tilde{T}}_{m}\left(1,0\right)\left(1-P_{m}^{false}\right)+{\tilde{T}}_{m}\left(1,1\right)P_{m}^{mis}\right)\right\}}{\left\{\delta \left(3\right)+\delta \left(4\right)\right\}}$$

(8) $$T_{m}\left(1,1\right)=\frac{\left\{\delta \left(3\right)\left({\tilde{T}}_{m}\left(0,0\right)P_{m}^{false}+{\tilde{T}}_{m}\left(0,1\right)\left(1-P_{m}^{mis}\right)\right)+\delta \left(4\right)\left({\tilde{T}}_{m}\left(1,0\right)P_{m}^{false}+{\tilde{T}}_{m}\left(1,1\right)\left(1-P_{m}^{mis}\right)\right)\right\}}{\left\{\delta \left(3\right)+\delta \left(4\right)\right\}}$$

### 2.3. Packet Arrival Process (PAP)

A finite queue of size Q packets is used at each CRV-SU CM to buffer packets. The CRV-SU CMs fetch packets from their finite queue for onward transmission to the CRV-SU CH. A batch Markovian process (see Equation (9)) is used to model the PAP of CRV-SUs with Y phases. Specifically, $P_{A}$ is used to denote the transition probability matrix of the PAP, as shown in Equation (9) below, for $A\in \left\{0,\;1,\;2,\;\cdots ,\;A_{m}\right\}$ arriving packets, with $A_{m}$ representing the maximum batch size:

(9) $$P_{A}=\left[\begin{array}{ccc} P_{A}\left(1,1\right) & \cdots & P_{A}\left(1,Y\right)\\ \vdots & & \vdots \\ P_{A}\left(Y,1\right) & \cdots & P_{A}\left(Y,Y\right) \end{array}\right]$$

With respect to Equation (9) above, $P_{A}\left(y,y^{\prime }\right)$ represents the probability that m data packets arrived at the finite queue with the phase changing from y to $y^{\prime }$. Correspondingly, the transition probability matrix P is given by $P=P_{0}+P_{1}+P_{2}+\cdots +P_{A_{m}}$. Let the steady state probability vector $\vec{\omega }$ of PAP be denoted by $\vec{\omega }={\left[\omega \left(1\right)\cdots \omega \left(y\right)\cdots \omega \left(Y\right)\right]}^{t}$. Then, the steady state probability that the phase of PAP is y is represented by the element ω(y) of the vector $\vec{\omega }$. Therefore, by solving ${\vec{\omega }}^{t}\left(\vec{1}\right)=1$ and ${\vec{\omega }}^{t}\left(P\right)={\vec{\omega }}^{t}$, this steady state probability vector $\vec{\omega }$ can be obtained. Accordingly, by weighting the probability of all phases with ω(y), the packet arrival rate (PAR) is obtainable through the following expression:

(10) $$\bar{\tau }=\overset{A_{m}}{\underset{A=1}{\sum }}A\left(\vec{\omega }{}^{t}P_{A}\right)\vec{1}.$$

### 2.4. SNO-CRAVNET Architecture

The model architecture and parameters of the SNO-CRAVNET scheme and a description of the initial resource allocation strategies of the scheme are presented in this subsection. Let the identically independent distributed (i.i.d.) subcarrier gain of CRV-SU n|n=1, 2, ⋯, L be represented by $a_{mn}$ on $m^{th}$ subcarrier, with $m=1,\;2,\;\cdots ,\;N_{C}$. Let $G_{mn}$ represent the complex circularly-symmetric Gaussian noise, and $G_{mn}\sim CN\left(0,\;\sigma_{\chi }^{2}\right)$, where $\sigma_{\chi }^{2}=B\left(N_{0}/N_{C}\right)$, with $N_{0}$ representing the noise density. Then, let the OFDM symbol transmitted by CRV-SU n over the $m^{th}$ subcarrier be denoted as $t_{mn}$, so that the OFDM symbol received at the destination can be expressed as $r_{mn}=\left(a_{mn}\times t_{mn}\right)+G_{mn}$. In the SNO-CRAVNET scheme, matrix $P_{N_{C}\times R}\left[O_{N_{C}\times R}\right]=\left[P_{mn}\right]$ denotes the transmit power allocation strategy, with the individual matrix elements represented by the instantaneous transmit power of CRV-SU n over channel m expressed as $P_{mn}=E\left[{\left|t_{mn}\right|}^{2}\right]$, where E[·] stands for the expected value operator. Additionally, matrix ${Ɽ}_{N_{C}\times R}\left[O_{N_{C}\times R}\right]=\left[R_{mn}\right]$ represents the rate allocation strategy, with the respective elements of the matrix denoted by the instant data-rate—$R_{mn}\left(P_{mn}\right)$—showing the total number of bits actually loaded on the $m^{th}$ subcarrier that is allocated to the CRV-SU n. Furthermore, the Multi-level Quadrature Amplitude Modulation (M-QAM) is used for the adjustment of the transmit power level, in agreement with the combined subcarrier power gains and the total number of loaded bits. Therefore, on each allocated CRV-SU n, the bit error rate (BER) according to Chung and Goldsmith [20] can be expressed as $BER_{mn}\approx 0.2\times exp\left\{-1.5\times \beta_{mn}/2^{\left[R_{mn}\left(P_{mn}\right)-1\right]}\right\}$, where $\beta_{mn}=P_{mn}{\left|a_{mn}\right|}^{2}/\sigma_{\chi }^{2}$ denotes the signal-to-noise ratio (SNR). By assuming, in this model, that the channel state information (CSI) [21,22] is known, we maximize the mutual information denoted as M(·) between the OFDM symbol transmitted by CRV-SU n over the $m^{th}$ subcarrier and the OFDM symbol received at the destination. Therefore, the maximum achievable channel capacity in a fading slot is represented as $M_{mn}^{C}\left(P_{mn}\right)=\max M\left(t_{mn}\;:\;r_{mn}|a_{mn}\right)=\log_{2}\left(1+P_{mn}{\left|a_{mn}\right|}^{2}\varphi \right)$, where $\varphi =-1.5/\left\{\ln\left(5\times BER_{mn}\right)\times \sigma_{\chi }^{2}\right\}$. Considering this, transmissions can only be successful, if and only if, $M_{mn}^{C}\left(P_{mn}\right)>R_{mn}\left(P_{mn}\right)$ (i.e., the maximum achievable capacity is greater than the instantaneous specified data-rate). Contrarily, when $M_{mn}^{C}\left(P_{mn}\right)=R_{mn}\left(P_{mn}\right)$, i.e., at the maximal point, according to Shannon’s theory, the feasible transmissions’ maximum instantaneous data-rate can be expressed as

(11) $$R_{mn}\left(P_{mn}\right)=\frac{B}{N_{C}}\log_{2}\left\{1+\left(P_{mn}{\left|a_{mn}\right|}^{2}\varphi \right)\right\},\;\forall m,\;n,$$

where $B/N_{C}$ represents the bandwidth of the respective dynamically available orthogonal subcarrier. Furthermore, the adaptive modulator ensures that the values of $R_{mn}\left(P_{mn}\right)$ are taken from set I={0, 1, 2, ⋯, I}, with I denoting the feasible maximum amount of information over each dynamically available orthogonal channel.

Additionally, in accordance with both the transmit power and rate allocation strategy, the channel allocation strategy is denoted by matrix $C_{N_{C}\times R}\left[O_{N_{C}\times R}\right]=\left[C_{mn}\right]$, where the channel allocation index signified by the matrix elements is represented by $C_{mn}\in \left\{0,\;1\right\}$. Therefore, $C_{mn}=1$ means that the dynamically available channel m is successfully allocated to CRV-SU n, and $C_{mn}=0$ means that no channel is allocated. Under SNO-CRAVNET architecture, two or more CRV-SUs cannot share a single channel at the same time. Therefore, a crucial constraint for the available channel allocation strategy is

(12) $$\overset{R}{\underset{n=1}{\sum }}C_{mn}\leq 1,\;\forall m,\;n.$$

Since the conditions of the available channel are random, in this paper, the expected value operator E[·] is used to indicate the random realization of CSI’s mean quantity (i.e., ${\left|a_{mn}\right|}^{2}$). Consequently, from Equations (11) and (12), the average data-rate of CRV-SU n can be expressed as

(13) $$R_{n}\left(C_{mn},\;P_{mn}\right)=E\left[\overset{N_{C}}{\underset{m=1}{\sum }}C_{mn}R_{mn}\left(P_{mn}\right)\right],\;\forall n.$$

Likewise, amongst all available channels and the CRV-SUs, the overall data-rate is given by

(14) $$\bar{R}\left(C_{mn},\;P_{mn}\right)=E\left[\overset{R}{\underset{n=1}{\sum }}\overset{N_{C}}{\underset{m=1}{\sum }}C_{mn}R_{mn}\left(P_{mn}\right)\right]$$

Therefore, to guarantee that the transmit power allocated to the CRV-SUs occupying every dynamically available orthogonal subcarrier does not exceed the target and is maintained below the average transmit power $P_{Tot.}$, available at the CR-BS, the condition for the transmit power allocation strategy is expressed as

(15) $$\overset{R}{\underset{n=1}{\sum }}\overset{N_{C}}{\underset{m=1}{\sum }}C_{mn}P_{mn}\leq P_{Tot.}\;$$

### 2.5. Interference Constraints

The regulations employed in the system model of SNO-CRAVNET to control interference against PUs’ transmission from CRV-SUs’ transmission are presented in this sub-section. In this model, $R^{\prime }$ PUs are considered in the network (i.e., the licensed users with ownership rights over the radio spectrum). On the contrary, when the CRV-SUs exploit the identified available spectrum holes for their own transmissions, they should do so in a fashion that ensures no harmful interference with the PUs with ownership rights over the spectrum band. Therefore, to guarantee the absolute avoidance of interference towards the PUs, CRV-SUs must strictly adhere to cognitive capabilities, which include, first and foremost, reliably intelligently sensing for the availability of spectrum holes to effectively confirm whether the channel is idle or currently occupied by a licensed owner. Secondly, upon confirming the existence of spectrum holes, the CRV-SUs should intelligently change their radio parameters for efficient exploitation of the identified spectrum holes, without causing interference to any ongoing transmissions of the PUs.

In Section 2.4, it is stated that under the SNO-CRAVNET scheme, each communication channel can only be allocated to a single CRV-SU at a time. Despite the allocation of one channel to one CRV-SU at a time, the communication quality of the channel, to a large extent, also affects the communications of the CRV-SUs. Therefore, the communication quality of the channel must be maintained by ensuring that the signal-to-interference-and-noise ratio (SINR) of the CRV-SU n is not lower than a predetermined threshold value $\beta_{n}^{min}.$ An acceptable QoS condition (Due to the orthogonality of the channels/subcarriers, the resulting interference between CRV-SUs is ignored, as is shown in Equation (12)) is obtained and expressed as

(16) $$\beta_{n}^{min}\leq E\left[\overset{N_{C}}{\underset{m=1}{\sum }}C_{mn}P_{mn}{\left|a_{mn}\right|}^{2}\right]/\sigma_{\chi }^{2}.\;$$

Therefore, Equation (16) can be expressed in a simplified form as

(17) $${Ᵽ}_{n}^{min}\leq E\left[\overset{N_{C}}{\underset{m=1}{\sum }}C_{mn}P_{mn}{\left|a_{mn}\right|}^{2}\right],\;\forall n,$$

where ${Ᵽ}_{n}^{min}=\left(\sigma_{\chi }^{2}\times \beta_{n}^{min}\right)=B\left(N_{0}/N_{C}\right)\times \beta_{n}^{min}$.

Additionally, to guarantee the protection of possible transmissions from licensed users (i.e., PUs) of the spectrum band, at each ${n^{\prime }}^{\left(th\right)}$ PU, with $n^{\prime }=1,\;2,\;3,\;\cdots ,\;R^{\prime }$, the received SNIR must be greater than $\beta_{PU}^{min}$, where $\beta_{PU}^{min}$ represents the predetermined threshold value applied to protect any ongoing transmissions from PUs. Let the distance between the $n^{\prime }$th PU and CR-BS be given as $d_{n^{\prime }}^{CR-BS}$, so that another interference constraint to protect the PUs’ transmission can be given as

(18) $$\beta_{PU}^{min}\leq \frac{{\left(d_{n^{\prime }}^{CR-BS}\right)}^{-\upsilon }\times P_{n^{\prime }}^{PU}}{\left(B\times N_{0}^{n^{\prime }}+{\left(d_{nn^{\prime }}\right)}^{-\upsilon }\times \left(\sum_{m=1}^{N_{C}}C_{mn}P_{mn}\right)\right)},\;$$

where υ denotes the exponent of path attenuation and $P_{n^{\prime }}^{PU}$ is the $n^{\prime }$th PU’s transmit power. $d_{nn^{\prime }}$ represents the distance between *n*th CRV-SU and $n^{\prime }$th PU, while $N_{0}^{n^{\prime }}$ represents the noise spectral density (i.e., noise density) of the $n^{\prime }$th PU. With the help of Location-Based Systems (LBSs), for instance, the Global Positioning System (GPS), both distances, $d_{n^{\prime }}^{CR-BS}$ and $d_{nn^{\prime }}$ can be easily obtained. In addition, information on the CRV-SU’s features can be obtained by the CR-BS through feedback channels. Therefore, without a loss of generality, Equation (18) can be further simplified and expressed as

(19) $${Ᵽ}_{n}^{max}\geq \overset{N_{C}}{\underset{m=1}{\sum }}C_{mn}P_{mn},\;\forall n,$$

where ${Ᵽ}_{n}^{max}=\left(\left({\left(d_{n^{\prime }}^{CR-BS}/d_{nn^{\prime }}\right)}^{-\upsilon }\times P_{PU}/\beta_{PU}^{min}\right)-\left(B\times N_{0}^{n^{\prime }}/{\left(d_{nn^{\prime }}\right)}^{-\upsilon }\right)\right)$. From Equation (19), the stipulated condition guarantees that the potential transmissions of the PU are fully protected if and only if the CRV-SU n’s total transmit power is constrained over channel n by the predefined threshold ${Ᵽ}_{n}^{max}$.

## 3. The Utility of SNO-CRAVNET and Problem Formulation

The design methodology of the SNO-CRAVNET’s objectives with its SNO-CRAVNET game is presented in this section in the form of a convex optimization problem, with its associated players represented by the ℝ CRV-SUs. The design of the game bargaining scheme methodologies for the CR-enabled IoV network system is proposed in this section. We assume that each ℝ CRV-SU, for instance, CRV-SU n, has an initial utility $U_{n}^{0}\geq 0$, which represents its acceptable minimum QoS constraint with respect to the data-rate and the corresponding utility function $f_{n}$. Under the symmetric Nash bargaining (SNB) theory, each utility function $f_{n}$ is usually designated as a convex and closed subset of $F^{R}=\left\{℧\right\}$, with $F^{R}$ and ℧ denoting the set of game theory strategies of the ℝ CRV-SU players and utility vectors’ space, respectively. Let us assume that $U_{n}^{0}$ is conveniently achievable for all the ℝ CRV-SU players. Then, it follows that at least a feasible subspace ${℧}_{0}$ exists in ℧, so that the utility vector, for instance, $f\left(\omega \right)=\left\{f_{1},\;f_{2},\;f_{3},\cdots ,\;f_{\mathbb{R}}\right\}$, becomes equal or bigger in comparison to the initial utility vector, such as, $U^{0}=\left\{U_{1}^{0},\;U_{2}^{0},U_{3}^{0},\;\cdots ,\;U_{\mathbb{R}}^{0}\right\}$. Therefore, the subset ${℧}_{0}$ as the element of ℧ can be expressed as ${℧}_{0}=\left\{\omega \in ℧|f\left(\omega \right)\geq U^{0}\right\}$. Additionally, let us suppose that the set of utility that can be achieved is denoted by  Ʈ={f(ω)|ω∈℧} and the category of sets of utility policies that satisfies $U^{0}$, which is the minimum utility bound, is denoted as $B=\left[Ʈ,\;U^{0}|Ʈ\subset F^{\mathbb{R}}\right]$. Therefore, in accordance with the Symmetric NBS theory (see [23]), there exists a unique solution, for instance, ${Տ}_{nbs}|B⟶F^{\mathbb{R}}$, which satisfies the following axioms:

(a) ${Տ}_{nbs}\left(Ʈ,\;U^{0}\right)$ ensures a minimum utility guarantee, for instance, ${Տ}_{nbs}\left(Ʈ,\;U^{0}\right)\in {Ʈ}^{0}$, where ${Ʈ}^{0}=\left\{U\in Ʈ|U\geq U^{0}\right\}$, ∀n;
(b) ${Տ}_{nbs}\left(Ʈ,\;U^{0}\right)$ is the Pareto optimal, which implies that other allocations ${Տ}_{nbs}^{\prime }\left(Ʈ,\;U^{0}\right)$ capable of guaranteeing a higher performance for all the ℝ CRV-SUs simultaneously do not exist, that is, ${Տ}_{nbs}^{\prime }\left(Ʈ,\;U^{0}\right)<{Տ}_{nbs}\left(Ʈ,\;U^{0}\right),\;\exists n$ and ${Տ}_{nbs}^{\prime }\left(Ʈ,\;U^{0}\right)\leq {Տ}_{nbs}\left(Ʈ,\;U^{0}\right),$ ∀n;
(c) ${Տ}_{nbs}\left(Ʈ,\;U^{0}\right)$ guarantees symmetry, which implies that all the ℝ CRV-SUs have equal priorities, for instance, supposing that Ʈ is symmetric with regards to a sub-set Q⊆{1, 2, 3, ⋯, n, ⋯, ℝ} and U∈Ʈ, n, $n^{\prime \prime }\in Q$ so that $U_{n}^{0}=U_{n^{\prime \prime }}^{0}$ implies that ${Տ}_{nbs}{\left(Ʈ,\;U^{0}\right)}_{n}={Տ}_{nbs}{\left(Ʈ,\;U^{0}\right)}_{n^{\prime \prime }}$, $n\neq n^{\prime \prime }$;
(d) ${Տ}_{nbs}\left(Ʈ,\;U^{0}\right)$ guarantees fairness by maintaining the independence of irrelevant alternatives, for instance, if the feasible set decreases and the solution keeps on being feasible, it follows that the solution for the lesser achievable set remains the same point. It can be expressed as ɰ⊂Ʈ, $\left(ɰ,\;U^{0}\right)\in B$ and ${Տ}_{nbs}\left(Ʈ,\;U^{0}\right)\in B$, then ${Տ}_{nbs}\left(Ʈ,\;U^{0}\right)={Տ}_{nbs}\left(ɰ,\;U^{0}\right)$, ∀n.

Without a loss of generality, the property of the SNO-CRAVNET is described using the following theorem.

**Theorem 1**. *It is assumed that the utility function defined by*℧*is convex upper bounded. Therefore,*℧*is convex and equal to*$℧\subseteq F^{\mathbb{R}}$*. Then, it is supposed that*Ɲ*is the set of indices of ℝ CRV-SUs that are capable of achieving a strictly superior performance in comparison to their initial performance. Therefore, it follows that there exists a symmetric Nash bargaining point*ω*, which confirms*$f_{n}\left(\omega \right)\geq U_{n}^{0}$*,*n∈Ɲ*and consists of a unique solution for the maximization problem expressed below:*

(20) $$\max\underset{n\in Ɲ}{\prod }\left(f_{n}\left(\omega \right)-U_{n}^{0}\right),\;\omega \in {℧}_{0}.$$

**Proof.** Theorem 1’s Proof is similar to that of the SNBS feature provided in [24] (Proof omitted here because it is similar to the one in [24] and also due to space limitations). □

Irrespective of the fact that the logarithmic basis of the optimization objective in Equation (20) stands, it is observed that resource allocation mechanisms (i.e., allocation problems) which depend on Theorem 1 are not typically convex over given convex sets. In particular, with such allocation problems under certain constraints, the convexity and existence of the feasible set which can satisfy the objective and all the constraints have to be thoroughly investigated. For instance, with respect to the CR constraints on transmit power allocation policy, channel selection, stability (i.e., protection of PU’s communication), and SNIR, as shown in Equations (12), (15), (17) and (19), the throughput definition given by Equation (13) can be adopted as the optimization objective in Equation (20) above. Therefore, an initial SNO-CRAVNET problem can be expressed as follows: Find the joint optimal transmit power and subcarrier allocation strategies ${\mathbb{C}}_{N_{C}\times \mathbb{R}}\left[O_{N_{C}\times \mathbb{R}}\right]$ and ${\mathbb{P}}_{N_{C}\times \mathbb{R}}\left[O_{N_{C}\times \mathbb{R}}\right]$.

(21) $$\underset{\mathbb{C},\;\mathbb{P}\;}{\max}E\left(\underset{Ʈ\left({\mathbb{C}}_{mn},P_{mn}\;\right)}{\underbrace{\overset{\mathbb{R}}{\underset{n=1}{\sum }}\ln\left(\left(\overset{N_{C}}{\underset{m=1}{\sum }}{\mathbb{C}}_{mn}R_{mn}\left(P_{mn}\right)\right)-U_{n}^{0}\right)}}\right),\;$$

subject to

(22) $$C_{mn}\in \left\{0,\;1\right\},\;\forall m,n,$$

(23) $$\overset{\mathbb{R}}{\underset{n=1}{\sum }}C_{mn}\leq 1,\;\forall m,n,$$

(24) $$P_{mn}\geq 0,\;\forall m,n,$$

(25) $$\overset{\mathbb{R}}{\underset{n=1}{\sum }}\overset{N_{C}}{\underset{m=1}{\sum }}C_{mn}P_{mn}\leq P_{Tot.},\;\forall m,n,$$

(26) $$E\left[\overset{N_{C}}{\underset{m=1}{\sum }}C_{mn}P_{mn}{\left|a_{mn}\right|}^{2}\right]\geq P_{n}^{min},\;\forall n,$$

(27) $$\overset{N_{C}}{\underset{m=1}{\sum }}C_{mn}P_{mn}\leq P_{n}^{max},\;\forall n.$$

The problem shown in Equation (21) and in the constraints (22)–(27) is a mixed combinatorial problem because it includes both a discrete variable $\left\{C_{mn}\right\}$ and continuous variable $\left\{P_{mn}\right\}$. Generally, the conventional approach normally adopted to solve such a mixed combinatorial problem is usually applied by performing an exhaustive search method [25] over the ℝ CRV-SUs and $N_{C}$ number of dynamically available channels. Therefore, there are a total of ${\mathbb{R}}^{N_{C}}$ possible channel assignments. To guarantee that the individual requirement for each of the ℝ CRV-SUs is satisfied for each of the ${\mathbb{R}}^{N_{C}}$ possible channel assignments, the total transmit power $P_{Tot}$ is allocated and, at the same time, summation of the SNO-CRAVNET data-rate of each of the ℝ CRV-SUs is equally maximized, accordingly.

Consequently, while all the constraints in Equations (22)–(27) are satisfied, the assignment of the dynamically available channels, together with their corresponding total transmit power allocation $P_{Tot.}$ which leads to the biggest summation of the data-rate, becomes the overall optimal solution. However, because of the high computational complexity of this method [25], together with the known limited computation, bandwidth, and storage resources in vehicular communication networks [26,27], extremely complex algorithms cannot be the best alternative for implementation in CR-enabled vehicular networks.

To overcome this challenge, the mixed combinatorial problem seen in Equation (21) and in the constraints shown in Equations (22)–(27) is methodically transformed to a convex optimization problem. The key aim of this transformation of the mixed combinatorial problem into a convex optimization problem is to make sure that the outcome of the transformation process must be a new problem that can symmetrically embrace the property of the proposed SNO-CRAVNET under the regulations of the emerging CR system. Furthermore, the new convex optimization problem must be defined over a feasible set that maintains its convexity and, at the same time, ensures that all the involved constraints are satisfied. The process of the transformation is as shown below. Firstly, as presented in Section 2.4, the requirement $R_{mn}\left(P_{mn}\right)\in \;I$ is relaxed into $R_{mn}\left(P_{mn}\right)\in \left[0,\;I\right]$, so that $R_{mn}\left(P_{mn}\right)$ can become a real number between the interval [0, I]. From Equation (12), apart from the discrete $\left\{C_{mn}\right\}$ variables, a set of new real-valued ${\tilde{C}}_{mn}$ variables between the interval [0, 1] is introduced, for instance, ${\tilde{C}}_{mn}\in \left[0,\;1\right]$. In particular, in accordance with the study of Wong et al. [28], ${\tilde{C}}_{mn}$ can be considered as a time-sharing factor of the *m*th subcarrier, which shows the period of time that subcarrier m is allocated to CRV-SU n over every one of the transmission frames. Then, with the aid of the time-sharing transformation, the objective $Ʈ\left({\tilde{C}}_{mn},\;P_{mn}\right)$ can be defined as convex over ${\tilde{C}}_{mn}$, though it still remains non-convex over $\left({\tilde{C}}_{mn},\;P_{mn}\right)$. Secondly, with the help of the same time-sharing approach, $P_{mn}$ is transformed into a continuous variable ${\tilde{P}}_{mn}=P_{mn}{\tilde{C}}_{mn}$, ∀m,n, which yields ${\tilde{P}}_{mn}\in \left[0,I\cdot {\tilde{C}}_{mn}\;\right]$. Therefore, with ${\tilde{C}}_{mn}$ and ${\tilde{P}}_{mn}$, the reformulated convex optimization problem can now easily be formulated: Find the optimal joint channel and transmit power allocation strategies ${\tilde{\mathbb{C}}}_{N_{C}\times \mathbb{R}}\left[O_{N_{C}\times \mathbb{R}}\right]$ and ${\tilde{\mathbb{P}}}_{N_{C}\times \mathbb{R}}\left[O_{N_{C}\times \mathbb{R}}\right].$

(28) $$\underset{\tilde{\mathbb{C},}\;\tilde{\;\mathbb{P}}\;}{\max}E\left(\underset{Ʈ\left({\tilde{C}}_{mn},\;{\tilde{P}}_{mn}\;\right)}{\underbrace{\overset{\mathbb{R}}{\underset{n=1}{\sum }}\ln\left(\left(\overset{N_{C}}{\underset{m=1}{\sum }}{\tilde{C}}_{mn}R_{mn}\left({\tilde{C}}_{mn},\;{\tilde{P}}_{mn}\right)\right)-U_{n}^{0}\right)}}\right),\;$$

subject to

(29) $${\tilde{C}}_{mn}\in \left[0,\;1\right],\;\forall m,n,$$

(30) $$\overset{\mathbb{R}}{\underset{n=1}{\sum }}{\tilde{C}}_{mn}\leq 1,\;\forall m,n,$$

(31) $${\tilde{P}}_{mn}\geq 0,\;\forall m,n,$$

(32) $$\overset{\mathbb{R}}{\underset{n=1}{\sum }}\overset{N_{C}}{\underset{m=1}{\sum }}{\tilde{P}}_{mn}\leq P_{Tot.},$$

(33) $$E\left[\overset{N_{C}}{\underset{m=1}{\sum }}{\tilde{P}}_{mn}{\left|a_{mn}\right|}^{2}\right]\geq P_{n}^{min},\;\forall n,$$

(34) $$\overset{N_{C}}{\underset{m=1}{\sum }}{\tilde{P}}_{mn}\leq P_{n}^{max},\;\forall n.$$

Between Equations (29)–(34), the constraints presented in Equations (29) and (30) guarantee that, at a given time-share, only one CRV-SU can be allocated a channel and must adhere to the properties of Equation (12) (see Section 2.4). The constraint in Equation (31) guarantees that the allocated transmit power must not be negative, while the constraint provided in Equation (32) maintains the transmitted power, in order to ensure that the transmit power allocated to the CRV-SU n occupying all the dynamically available orthogonal channels is maintained below the total transmit power $P_{Tot.}$ available at CR-BS, as defined in Equation (15). Lastly, as illustrated in Equations (17) and (19), transmit power constraints for each ℝ CRV-SU and PU are guaranteed by constraints presented in Equations (33) and (34), respectively.

**Proposition 1**. *In the above stated optimal joint subcarrier and transmit power allocation strategies, the problem defined in Equation (28) and in the constraints presented in Equations (29)–(34) is a convex optimization problem.*

**Proof.** Proposition 1’s Proof is shown in Appendix A. □

With regard to Proposition 1, it is established that the problem defined in Equation (28) and in the constraints presented in Equations (29)–(34) is clearly convex over a given convex set. Therefore, there exists a unique optimal solution that can be achieved within the polynomial time [25].

**Proposition 2.***Let us assume that*${\tilde{P}}_{mn}>0$*. Then,*$Ʈ\left({\tilde{C}}_{mn},\;{\tilde{P}}_{mn}\right)$*, as shown in Equation (28), can stringently increase for all*${\tilde{C}}_{mn}$*, thereby satisfying*${\tilde{C}}_{mn}R_{mn}\left({\tilde{C}}_{mn},\;{\tilde{P}}_{mn}\right)>U_{n}^{0}$.

**Proof.**Appendix A presents the Proof of Proposition 2. □

Proposition 2 certifies that the transformation of the objective in Equation (20), as well as in Equation (21) to $Ʈ\left({\tilde{C}}_{mn},\;{\tilde{P}}_{mn}\right)$ in Equation (28), can be achieved by exploiting the firmly increasing property of the logarithm function.

**Proposition 3.** *The utility function* $Ʈ\left({\tilde{C}}_{mn},\;{\tilde{P}}_{mn}\right)$ *proposed in Equation (28) is Nash bargaining theorem compliant and, at the same time, satisfies the proportional fairness metric.*

**Proof.**Appendix A presents the Proof of Proposition 3. □

In our case, Proposition 3 shows that, for the data-rate allocation, a unique Nash bargaining equilibrium can be obtained. Likewise, as a special case of the NBS fairness [29], proportional fairness can be achieved when $U_{n}^{0}=0$, ∀n.

## 4. Optimal Resource Scheduling Strategies

The convex optimization problem’s optimal solution, which is presented in Equation (28) and in the constraints expressed in Equations (29)–(34), is derived in this section. Additionally, a simple and efficient strategy, which supports an iteration-independent joint transmit power and subcarrier scheduling, is proposed. The optimal subcarrier allocation ${\tilde{C}}_{mn}$ with a consideration of the time-sharing approach is a real number implying the fraction of time which subcarrier m requires for the transmission of a given amount of information. Firstly, uniform transmit power scheduling, that is, ${\tilde{P}}_{mn}=P_{Tot.}/\left(N_{C}\cdot B\right)$, is performed for all the available subcarriers. Then, an equal amount of information is transferred over all the available subcarriers. Secondly, based on the study carried out by Hahne [29], the optimal time-sharing subcarrier scheduling strategy is obtained.

**Theorem 2.***The SNO-CRAVNET optimal time-sharing subcarrier scheduling strategy is given as*${\tilde{\mathbb{C}}}_{N_{C}\times \mathbb{R}}^{*}\left[O_{N_{C}\times \mathbb{R}}\right]=\left[{\tilde{\mathbb{C}}}_{mn}^{*}\right]$*, and the individual matrix elements are expressed as*

(35) $${\tilde{\mathbb{C}}}_{mn}^{*}={ƛ}_{mn}^{-1}\left({₼}_{m}^{*}\right),$$

*where*${₼}_{m}^{*}={ɸ}^{-1}\left(1\right)$, ∀m.

**Proof.**Appendix B presents the Proof of Theorem 2. □

Based on Equation (35), the matrix ${\tilde{\mathbb{C}}}_{N_{C}\times \mathbb{R}}^{*}\left[O_{N_{C}\times \mathbb{R}}\right]=\left[{\tilde{\mathbb{C}}}_{mn}^{*}\right],$ which illustrates the time-sharing scheduling of each subcarrier for all the ℝ CRV-SUs, is determined. Additionally, this further helps in determining the quality of each subcarrier, that is, based on Equation (35), if it is observed that ${\tilde{\mathbb{C}}}_{mn}^{*}<{\tilde{\mathbb{C}}}_{m^{\prime }n}^{*},$ this indicates that even though both subcarriers were allocated an equal amount of transmit power, subcarrier m requires less time than subcarrier $m^{\prime }$ for the transfer of an equal amount of information by the same CRV-SU n. Therefore, subcarrier m is in better conditions, i.e., has a higher quality in comparison to subcarrier $m^{\prime }$. Furthermore, accounting for ${\tilde{\mathbb{C}}}_{N_{C}\times \mathbb{R}}^{*}\left[O_{N_{C}\times \mathbb{R}}\right]$, the optimal transmit power scheduling strategy is defined as shown here.

**Theorem 3.***The SNO-CRAVNET optimal transmit power scheduling strategy is given by*${\tilde{\mathbb{P}}}_{N_{C}\times \mathbb{R}}^{*}\left[O_{N_{C}\times \mathbb{R}}\right]=\left[{\tilde{P}}_{mn}^{*}\right]$*and the individual matrix elements are given by*

(36) $${\tilde{P}}_{mn}^{*}=\frac{{\tilde{\mathbb{C}}}_{mn}^{*}}{{\left|a_{mn}\right|}^{2}\varphi }{\left\{{Þ}_{mn}\cdot \exp\left(w\left(\ln\left(2^{\left(\frac{X_{mn}\cdot N_{C}}{B\cdot {\tilde{\mathbb{C}}}_{mn}^{*}\cdot {Þ}_{mn}}\right)}\right)\right)\right)-1\right\}}^{+},$$

*where*w(·)*represents the Lambert*w*-function. The definitions of both*${Þ}_{mn}$*and*$X_{mn}$*are contained in Appendix B, and the symbol*${\left(z\right)}^{+}$*represents*max (0, z).

**Proof.**Appendix B presents the Proof of Theorem 3. □

The optimal transmit power scheduling for CRV-SU n on every subcarrier m is obtained from Theorem 3. In other words, the optimal transmit power that subcarrier m requires to be able to transmit a given amount of information based on the licensed PU’s protection parameters, the associated subcarrier’s conditions, and the characteristics of CRV-SU n is denoted by ${\tilde{P}}_{mn}^{*}$.

Consequently, through a linear search of the $N_{C}$ subcarriers, efficient resource scheduling can be performed, for instance, for m=1 to $N_{C}$, find the optimal CRV-SU $n^{*}=\arg\min{\tilde{\mathbb{C}}}_{mn}^{*}$. Then, allocate the corresponding transmit power as defined in Equation (36) to all the selected CRV-SUs $n^{*}$s. Despite the fact that the procedure derives ${\tilde{\mathbb{C}}}_{mn}^{*}$s accounting for the subcarrier scheduling constraints presented in Equations (29) and (30) under the symmetric NBS’s rule, the procedure indirectly considers the transmit power constraints presented in Equations (31)–(34). Then, with high QoS heterogeneity amongst the ℝ CRV-SUs (i.e., concerning the subcarrier’s stringent QoS requirements and interference conditions), ${\tilde{\mathbb{C}}}_{mn}^{*}<{\tilde{\mathbb{C}}}_{m^{\prime }n}^{*}$ does not necessarily indicate that ${\tilde{P}}_{mn}^{*}<{\tilde{P}}_{m^{\prime }n}^{*}$, and vice versa, which leads to transmit power inefficiency. Therefore, to overcome this, the following optimal transmit power scheduling method is introduced, in order to increase the transmit power efficiency.

**Theorem 4.***The SNO-CRAVNET optimal transmit power scheduling strategy is given as*${\mathbb{P}}_{N_{C}\times \mathbb{R}}^{*}\left[O_{N_{C}\times \mathbb{R}}\right]=\left[P_{mn}^{*}\right]$*, where the corresponding matrix elements are determined via searching amongst the dynamically available*$N_{C}$*subcarriers. For*m=1*to*$N_{C}$,

(37) $$n^{*}=\arg\min{\tilde{P}}_{mn}^{*},\;P_{mn}^{*}=\{\begin{array}{c} {\tilde{P}}_{mn}^{*},\;\mathrm{if}\;n=n^{*}\\ 0,\;\mathrm{otherwise} \end{array}$$

*where*$n^{*}$ represents the optimal CRV-SU.

**Proof.**Appendix B presents the Proof of Theorem 4. □

Using Equation (37), matrix ${\mathbb{P}}_{N_{C}\times \mathbb{R}}^{*}\left[O_{N_{C}\times \mathbb{R}}\right]$ is obtained, which indicates that the optimal transmit power is allocated to the optimal CRV-SU $n^{*}$ on subcarrier m. Therefore, the optimal transmit power of each CRV-SU can be determined through $P_{n}^{*}=\sum_{m=1}^{N_{C}}P_{mn}^{*}$, ∀n. Likewise, accounting for ${\mathbb{P}}_{N_{C}\times \mathbb{R}}^{*}\left[O_{N_{C}\times \mathbb{R}}\right]$, the optimal subcarrier allocation can be defined as shown here.

**Theorem 5.** *The SNO-CRAVNET optimal subcarrier allocation strategy is given by* ${\mathbb{C}}_{N_{C}\times \mathbb{R}}^{*}\left[O_{N_{C}\times \mathbb{R}}\right]=\left[{\mathbb{C}}_{mn}^{*}\right]$ *, where the individual matrix elements are determined by*

(38) $${\mathbb{C}}_{mn}^{*}=\{\begin{array}{c} 1,\;\mathrm{if}\;n=n^{*}\\ 0,\;\mathrm{otherwise} \end{array}$$

**Proof.**Appendix B presents the Proof of Theorem 5. □

Accordingly, from Equation (38), the optimal subcarrier scheduling matrix ${\mathbb{C}}_{N_{C}\times \mathbb{R}}^{*}\left[O_{N_{C}\times \mathbb{R}}\right]$ is obtained. Consequently, by using Equations (35)–(38), the joint transmit power and subcarrier allocation strategy for CRV-SU systems is determined as illustrated, with the aid of the pseudo-code, in Algorithm 1.

Additionally, from the combination of Equations (11) and (36)–(38), the optimal rate scheduling strategy ${Ɽ}_{N_{C}\times \mathbb{R}}^{*}\left[O_{N_{C}\times \mathbb{R}}\right]=\left[R_{mn}^{*}\left({\mathbb{C}}_{mn}^{*},{\tilde{\mathbb{C}}}_{mn}^{*},{\tilde{P}}_{mn}^{*}\right)\right]$ is obtained, where the instantaneous optimal data-rate as the individual matrix elements is determined by

(39) $$R_{mn}^{*}=\frac{B}{N_{C}}\cdot {\mathbb{C}}_{mn}^{*}\cdot \log_{2}\left\{{Þ}_{mn}\cdot \exp\left(w\left(\ln\left(2^{\left(\frac{X_{mn}\cdot N_{C}}{B\cdot {\tilde{\mathbb{C}}}_{mn}^{*}\cdot {Þ}_{mn}}\right)}\right)\right)\right)\right\}.$$

Therefore, from Equations (14) and (39), the overall optimal throughput of the SNO-CRAVNET system amongst all the ℝ CRV-SUs and subcarriers can be obtained, for instance, ${\tilde{R}}^{*}\left({\mathbb{C}}_{mn}^{*},{\tilde{\mathbb{C}}}_{mn}^{*},P_{mn}^{*}\right)=E\left[\sum_{n=1}^{\mathbb{R}}\sum_{m=1}^{N_{C}}{\mathbb{C}}_{mn}^{*}R_{mn}^{*}\left({\mathbb{C}}_{mn}^{*},{\tilde{\mathbb{C}}}_{mn}^{*},P_{mn}^{*}\right)\right]$.

Furthermore, by substituting ${\mathbb{C}}_{mn}^{*}$ and $P_{mn}^{*}$ in the transformed convex optimization problem presented in Equations (28)–(34), it can be observed that an upper-bound of the maximum SNO-CRAVNET overall system throughput defined as $E\left[{\bar{ℜ}}^{*}\right]$ can be obtained, where ${ℜ}^{*}$ represents the reachable (i.e., maximum) SNO-CRAVNET overall system throughput of subcarrier $n^{*}$. On the contrary, by substituting ${\mathbb{C}}_{mn}^{*}$ and $P_{mn}^{*}$ in the original optimization problem presented in Equations (21)–(27), a lower-bound of the reachable data-rate defined as $E\left[{\underline{ℜ}}^{*}\right]$ can be obtained. Without a loss of generality, let ${ℜ}^{\mathrm{Tot}.}$ be the total reachable data-rate obtained by a combinatorial search of the original optimization problem presented in Equations (21)–(27); then, $E\left[{\bar{ℜ}}^{*}\right]\geq E\left[{ℜ}^{\mathrm{Tot}.}\right]\geq E\left[{\underline{ℜ}}^{*}\right]$. Therefore, the difference that exists between the lower-bound $E\left[{\underline{ℜ}}^{*}\right]$ and the upper-bound $E\left[{\bar{ℜ}}^{*}\right]$ of the maximum SNO-CRAVNET overall system throughput indicates how far apart the proposed scheme is from the actual optimal solution. Consequently, based on the experimental results shown in Section 5.2.1, it is shown that, in the case of the proposed scheme, the gap that exists between the lower-bound $E\left[{\underline{ℜ}}^{*}\right]$ and the upper-bound $E\left[{\bar{ℜ}}^{*}\right]$ of the maximum SNO-CRAVNET overall system throughput is insignificantly small, for instance, smaller than 0.016%.
**Algorithm 1** Pseudo-code to find the joint optimal transmit power and subcarrier allocation solution**Procedure:****Input**: Maximization of the expected optimal number of dynamically available subcarriers acquired in the *m*th slot.**Output**: Obtained joint optimal transmit power and subcarrier allocation solution.Initialization:1: Set ${\tilde{P}}_{mn}=P_{Tot.}/\left(B.N_{C}\right)$, ∀m,n
    Step 12: Find ${\tilde{\mathbb{C}}}_{N_{C}\times \mathbb{R}}^{*}\left[O_{N_{C}\times \mathbb{R}}\right]=\left[{\tilde{\mathbb{C}}}_{mn}^{*}\right]$ based on Equation (35) 3: Find ${\tilde{\mathbb{P}}}_{N_{C}\times \mathbb{R}}^{*}\left[O_{N_{C}\times \mathbb{R}}\right]=\left[{\tilde{P}}_{mn}^{*}\right]$ based on Equation (36)    Step 2     Based on the minimum ${\tilde{P}}_{mn}^{*}$, perform resource scheduling:4: For m=1 to $N_{C}$
5:   $n^{*}=\arg\min{\tilde{P}}_{mn}^{*}$, $P_{mn}^{*}=\{\begin{array}{c} {\tilde{P}}_{mn}^{*},\;\mathrm{if}\;n=n^{*}\\ 0,\;\mathrm{otherwise} \end{array}$
6: End For    Step 37: Obtain ${\tilde{\mathbb{P}}}_{N_{C}\times \mathbb{R}}^{*}\left[O_{N_{C}\times \mathbb{R}}\right]$ and ${\tilde{\mathbb{C}}}_{N_{C}\times \mathbb{R}}^{*}\left[O_{N_{C}\times \mathbb{R}}\right]$ using Equations (37) and (38), respectively.

## 5. Performance Evaluation

### 5.1. Simulation Settings

As depicted in Figure 1, the co-existence of the cellular OFDMA-based overlay CR-Assisted Vehicular NETwork with the PU network scenario of seven CCs (i.e., $J=\left\{L_{1},\;L_{2},\;\cdots ,\;L_{7}\right\}$) is considered. In each of the seven CCs (i.e., locations), there are two shared wireless channels. As defined in Equation (4), in the shared wireless channel m, the activity of PUs is modeled by $T_{m}$ for m=1, 2. The simulation experiments use a system which consists of ℝ=10 CRV-SUs, ℝ=20 CRV-SUs, and $N_{C}=64$, with a total power $P_{Tot.}=3\;W$. In each CRV-SU node, the duration of the time-slot is 20 ms and the queue size Q is 20 packets. The radius of the CCs is 5 km and the average packet arrival rate follows a Poisson process, with $\bar{\tau }=0.5$ packets per time-slot. The average speed of the CRV-SUs is 50 km/h. The frequency selective fading subcarrier involves six independent Rayleigh fading multipaths with an exponential power delay profile (PDP) of 100 ns. The transmit power for each of the ℝ CRV-SUs is constrained by the threshold $P_{n}^{min}=0.5\;W$. The rest of the parameters used in the simulations with their set values are shown in Table 3.

### 5.2. Discussion of the Results

The performance evaluation of SNO-CRAVNET was carried out in comparison with existing relevant scheduling schemes for CR-Assisted Vehicular NETwork systems. The relevant reference schemes selected for the purpose of performance evaluation against SNO-CRAVNET were the Dependent Rounding-based Scheme (DR) [30], Pure Nash Equilibrium Search scheme (PNE-S) [31], and Cuckoo Search scheme (CS) with Multi-objective Optimization based on the Decomposition scheme (MOCS/D) [32]. To ensure that consistency and fairness were maintained regarding the comparisons of the proposed SNO-CRAVNET and reference schemes, derivation of the optimal strategies of DR, PNE-S, and MOCS/D was achieved through optimization problems involving the constraints of CR, as shown in Equations (7) and (9), and further system throughput optimization constraints for the minimal CRV-SU’s utility requirement $U_{n}^{0}$. For example, $U_{n}^{0}\leq E\left[\sum_{m=1}^{N_{C}}R_{mn}\left({\mathbb{C}}_{mn}^{*},P_{mn}^{*}\right)\right]$ for DR, while $U_{n}^{0}\leq E\left[\sum_{m=1}^{N_{C}}R_{mn}\left({\mathbb{C}}_{mn}^{*},\;{\tilde{\mathbb{C}}}_{mn}^{*},\;P_{mn}^{*}\right)\right]$ for PNE-S and MOCS/D. The cost functions which correspond to each of the scenarios are shown below:

DR: $\max E\left[\sum_{n=1}^{\mathbb{R}}\sum_{m=1}^{N_{C}}R_{mn}\left({\mathbb{C}}_{mn}^{*},P_{mn}^{*}\right)\right]$, PNE-S: $\max\underset{1\leq n\leq \mathbb{R}}{\min}E\left[\sum_{n=1}^{\mathbb{R}}\sum_{m=1}^{N_{C}}R_{mn}\left({\mathbb{C}}_{mn}^{*},{\tilde{\mathbb{C}}}_{mn}^{*},P_{mn}^{*}\right)\right]$, and MOCS/D: $\max E\left[\sum_{n=1}^{\mathbb{R}}\sum_{m=1}^{N_{C}}R_{mn}\left({\mathbb{C}}_{mn}^{*},{\tilde{\mathbb{C}}}_{mn}^{*},P_{mn}^{*}\right)\right]$.

#### 5.2.1. System Throughput Evaluation

**Figure 3:**
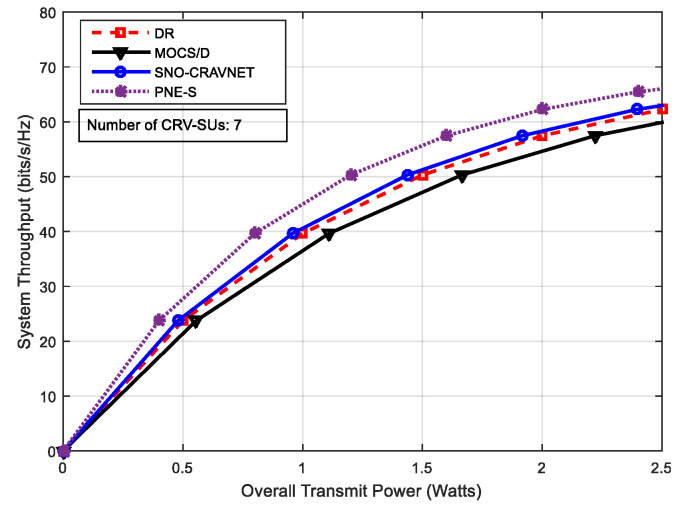
Performance evaluation using the achieved system throughput measured against the overall supplied transmit power with number of Cognitive Radio vehicular secondary users (CRV-SUs) = 7.

**Figure 4:**
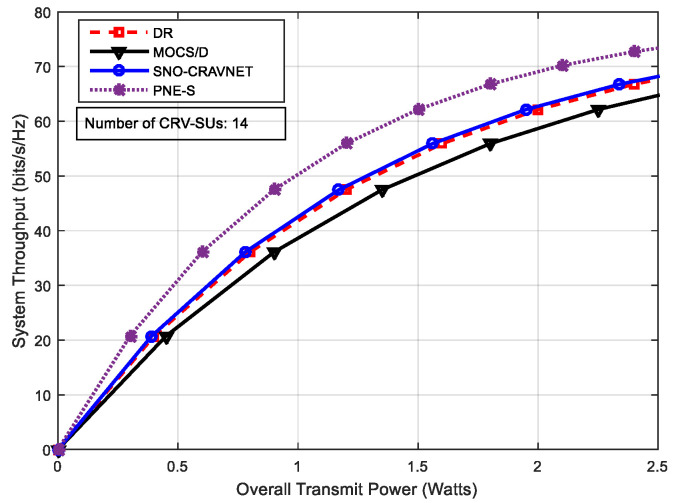
Performance evaluation using the achieved system throughput measured against the overall supplied transmit power with number of Cognitive Radio vehicular secondary users (CRV-SUs) = 14.

The performance of the proposed SNO-CRAVNET is depicted in Figure 3 through a comparison of the reference schemes DR, PNE-S, and MOCS/D, using the overall achieved system throughput measured against the overall supplied transmit power. The overall achieved average system throughput of each of the schemes, as expected, sharply increases with a corresponding increase in the total supplied transmit power. As can been seen in Figure 3 overleaf, the slightly higher overall achieved average system throughput of the PNE-S in comparison to the proposed SNO-CRAVNET is because PNE-S does not take into account the resource allocation fairness among the CRV-SUs, as opposed to SNO-CRAVNET, DR, and MOCS/D. On the other hand, although the performance of DR is nearly equal to that of the proposed SNO-CRAVNET and a little above the performance of MOCS/D, Figure 3 clearly shows that SNO-CRAVNET outperforms both. The same occurs in Figure 4, where the performance of all the schemes is seen to increase accordingly with a further increase in the number of CRV-SUs from 7 to 14. As is the case in Figure 3, SNO-CRAVNET still outperforms both DR and MOCS/D. This could be explained by the fact that DR requires additional transmit power in comparison to SNO-CRAVNET, whereas MOCS/D fails to utilize the subcarrier resources opportunistically, thereby resulting in a lower overall average system throughput performance, as can be seen in both Figure 3 and Figure 4 below.

#### 5.2.2. Average Throughput Gain Evaluation

**Figure 5:**
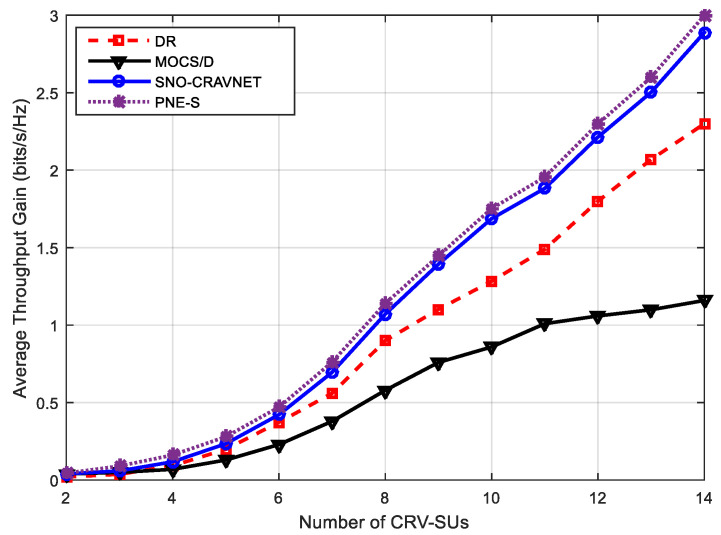
Performance evaluation using the overall achieved average throughput gain measured against the varying number of CRV-SUs.

In Figure 5 below, the performance evaluation of SNO-CRAVNET in comparison to DR, PNE-S, and MOCS/D using the overall achieved average throughput gain measured against the varying number of CRV-SUs is presented. Supposing the scheduler allocated an ${ℜ}_{Allocated}$ data-rate, the overall achieved average throughput gain could be calculated as ${ℜ}_{Allocated}-\sum_{n=1}^{\mathbb{R}}U_{n}^{0}$. As shown in Figure 5, the PNE-S obtained a slightly higher overall average throughput gain because of its non-fairness consideration among the ℝ CRV-SUs. In the case of SNO-CRAVNET, PNE-S only had a relatively marginal higher average throughput gain. For instance, when ℝ=6, the PNE Search scheme achieved an average throughput gain of 0.48 bits/s/Hz, whereas the proposed SNO-CRAVNET achieved 0.46 bits/s/Hz. Therefore, even though PNE-S does not consider fairness, it only outperformed the proposed fairness-considerate SNO-CRAVNET by 0.02 bits/s/Hz. However, in comparison to other scheduling schemes such as DR and MOCS/D that consider fairness, the proposed SNO-CRAVNET recorded a significantly higher overall average throughput gain, as can be seen in Figure 5. For example, when ℝ=14, SNO-CRAVNET achieved a value that was 0.5 bits/s/Hz and 1.6 bits/s/Hz higher than that of DR and MOCS/D, respectively.

#### 5.2.3. Transmit Power Gain Evaluation

**Figure 6:**
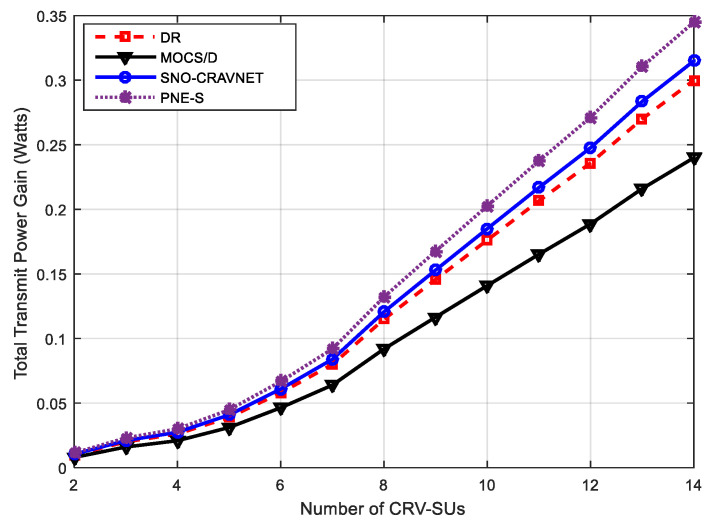
Performance evaluation using the total transmit power gain measured against a varying number of CRV-SUs.

Figure 6 demonstrates the performance evaluation of the proposed SNO-CRAVNET against the existing related schemes using the total transmit power gain measured against a varying number of CRV-SUs. Based on the assumption that $P_{n}^{min}$ and ${Ᵽ}_{Allocated}$ are the minimum power required by a CRV-SU and the minimum power required by a scheduler to guarantee the QoS requirements of each ℝ CRV-SU, the total transmit power gained is obtained as ${Ᵽ}_{Allocated}-\sum_{n=1}^{\mathbb{R}}P_{n}^{min}$. Figure 6 shows that SNO-CRAVNET achieves remarkably higher transmit power gain as the number of CRV-SUs increases compared with DR and MOCS/D. For instance, when ℝ=8, SNO-CRAVNET achieved 0.01 *W* and 0.04 *W* of total transmit power gain more than DR and MOCS/D, respectively. Similarly, when ℝ=14, SNO-CRAVNET achieved 0.02 W and 0.08 W of total transmit power gain more than DR and MOCS/D, respectively.

#### 5.2.4. Jain’s Fairness Index (JFI) Evaluation

**Figure 7:**
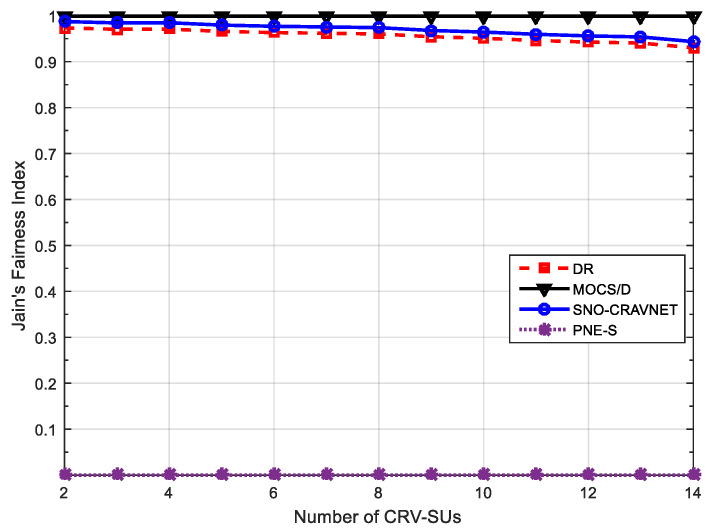
Resource allocation fairness performance evaluation using Jain’s fairness index (JFI) measured against a varying number of CRV-SUs.

The resource fairness provision was investigated, as depicted in Figure 7, through performance evaluation using JFI measured against a varying number of CRV-SUs. According to [2,33], the JFI is expressed as

(40) JFI=(∑n=1ℝ(ℜnUn0))2/(ℝ·(∑n=1ℝ(ℜnUn0)2)),

where ${ℜ}_{n}$ denotes CRV-SU n’s rate allocation. Consequently, JFI=1 indicates perfectly fair resource allocation by the scheduler. Conversely, JFI reduces towards 0 with an increase in the CRV-SUs rate’s disparity. In Figure 7, it can be seen that MOCS/D achieved perfectly fair resource allocation (i.e., JFI=1) due to non-opportunistic scheduling, but recorded a low overall average system throughput performance, as can be observed in both Figure 3 and Figure 4, and high transmit power demands. In contrast, as expected, the fairness inconsiderate PNE-S is the most unfair amongst the schemes and achieved the most imperfectly fair resource allocation (i.e., JFI=0) when 2≤ℝ≤14. However, Figure 7 demonstrates that both SNO-CRAVNET and DR can achieve fair resource allocation. Although it can be observed that their performances decrease with an increase in the number of CRV-SUs, SNO-CRAVNET continuously outperformed DR in all cases, (i.e., 2≤ℝ≤14). Accordingly, DR is forced to allocate resources less fairly in comparison to SNO-CRAVNET due to resource starvation. In general, SNO-CRAVNET shows a performance gain (nearly 5% improvement) over DR, as is evident in Figure 7, in terms of the percentage of JFI achieved. Obviously, in the case of both SNO-CRAVNET and DR, when the allocated transmit power is insufficient, the JFI value decreases.

#### 5.2.5. Accuracy of the Proposed Method

**Figure 8:**
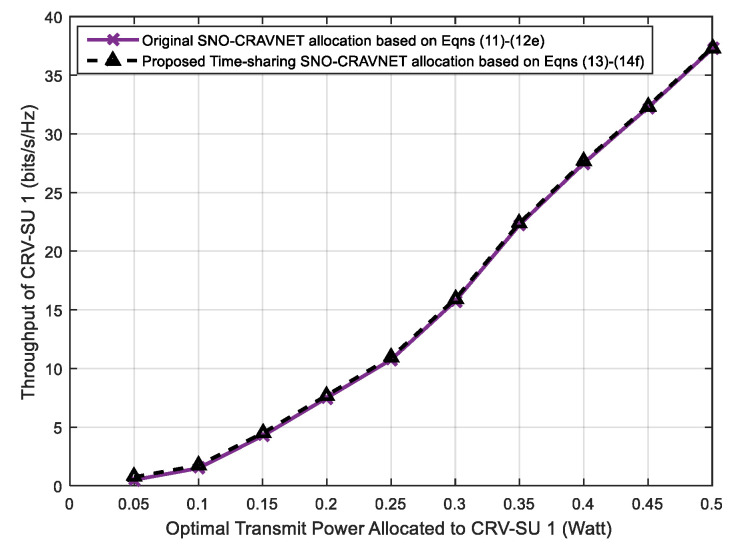
Performance evaluation using optimal throughput measured against the optimal supplied transmit power for CRV-SU 1.

**Figure 9:**
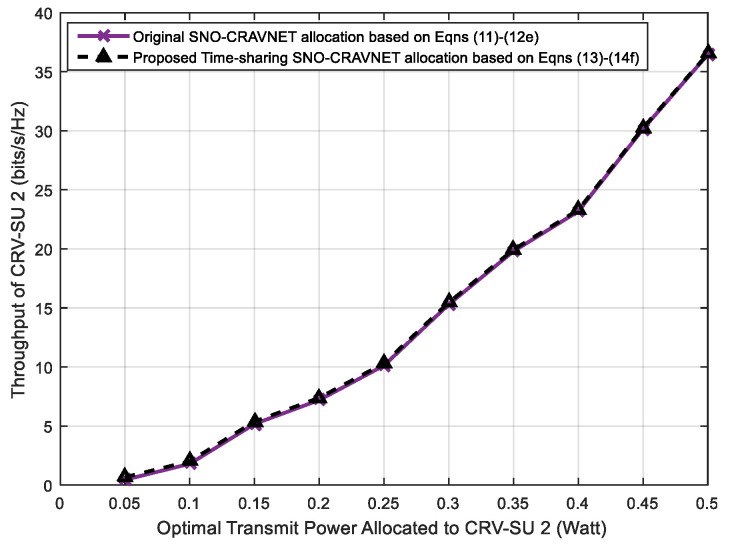
Performance evaluation using optimal throughput measured against the optimal supplied transmit power for CRV-SU 2.

The accuracy of the proposed time-sharing approach is studied in Figure 8 and Figure 9, which depict comparisons of the derived optimal strategies through a combinatorial search within the original optimization problem expressed in Equations (11) and (22)–(27) and the reformulated convex optimization problem presented in Equations (13) and (29)–(34) as discussed in Section 3, respectively. The comparisons were performed with respect to (*wrt*) the optimal supplied transmit power and achievable optimal throughput of CRV-SU 1 (see Figure 8) and CRV-SU 2 (see Figure 9). Furthermore, for other CRV-SUs, similar results were obtained. As clearly demonstrated in Figure 8 and Figure 9, the transmit power and the achievable optimal throughput values obtained in the case of CRV-SU 1 (see Figure 8) and CRV-SU 2 (see Figure 9) are nearly the same and the performance gaps in both cases are infinitesimally trivial, such as 0.014%. Therefore, both Figure 8 and Figure 9 show that the proposed time-sharing SNO-CRAVNET allocation based on Equations (13) and (29)–(34) is capable of achieving up to an average of 99.987% for the total theoretical capacity.

## 6. Conclusions

This paper has presented an efficient joint optimal subcarrier and transmit power allocation framework with QoS guarantee to support enhanced packet transmission over a Cognitive Radio-enabled IoV network system. The study proposed a novel SNBS-based wireless radio resource scheduling scheme in an OFDMA CR-enabled IoV network system. The CRV-SUs form clusters, leading to an improved CR-enabled IoV communication efficiency in a network system over the shared wireless radio channels (i.e., the channels that belong to licensed PUs). Although the shared wireless radio channels are primarily allocated to the PUs, the same channels can be opportunistically accessed by the CRV-SUs on the condition that the SINR with the PUs is maintained below the threshold level. Furthermore, a convex optimization problem was formulated by applying a time-sharing technique. The formulated convex optimization problem involves constraints on CR technology regulations, joint optimal subcarrier, and transmit power allocation. Then, the optimal subcarrier and transmit power allocation strategies were derived via mathematical analysis. The developed iteration-independent and low-complexity algorithm ensures easy convergence to Pareto optimality. Theoretical analysis and simulation results show that the proposed SNO-CRAVNET outperformed the reference scheduler schemes. In comparison to other scheduling schemes that are fairness-considerate, the proposed SNO-CRAVNET recorded a significantly higher overall average throughput gain, as is shown in Figure 5. Similarly, the accuracy of the proposed time-sharing method wrt the optimal transmit power and the achievable optimal throughput of CRV-SU 1 and CRV-SU 2 was investigated. It is shown in Figure 6 that the proposed time-sharing SNO-CRAVNET allocation based on the reformulated convex optimization problem is capable of achieving up to an average of 99.987% for the total theoretical capacity. In the same vein, the proposed SNO-CRAVNET scheme outperformed the other reference scheduling schemes in terms of fair resource allocation, which further emphasizes that the open issue of joint optimal subcarrier and transmit power allocation with QoS guarantee for enhanced data transmission over CR-IoVs was achieved.

An investigation of the integration of spectrum sensing in interweave-based CR-enabled IoV network systems represents an interesting possible future research direction. Under the interweave-based CR-enabled IoV network systems, the spectrum sensing accuracy remains an open issue due to prevailing sensing errors over wireless channels. Additionally, as part of future work, a hidden CRV-SU problem will be considered in deriving the transition probability matrix, in order to further understand how the presence of hidden CRV-SUs may affect the transition probability matrix and transmit power allocation.

## Figures and Tables

**Table 1:** List of key mathematical notations.

Symbol	Description
ℕ	Number of CR base stations
ℝ	Number of CRV-SUs in vehicular cluster formations
B	Channel bandwidth
$P_{n}^{max}$	Power threshold
$BN_{0}^{n^{\prime }}$	PU noise density
$d_{n^{\prime }}^{CR-BS}$	Distance between *n*′th PU and CR-BS
$d_{nn^{\prime }}$	Distance between *n*th CRV-SU and the *n*′th PU
$\beta_{PU}^{min}$	Interference constraint to protect the PUs’ transmissions
υ	Exponent of path attenuation
$BN_{0}$	Noise density
$P_{Tot.}$	Average transmit power available at the CR-BS
${\tilde{T}}_{m}$	State transition of shared channel m
$\left[P_{mn}\right]$	Transmit power allocation strategy
$N_{C}$	Number of orthogonal channels
Ɱ	Transition rate matrix
|J|	Cardinalty of set J
$ɱ\left(j,\;j^{\prime }\right)$	Rate at which the CRV-SUs’ cluster formation changes from ${𝓁}_{j}$ to ${𝓁}_{j^{\prime }}$ location
$\bar{\tau }$	Packet arrival rate
P	Transition probability matrix
$\vec{\omega }$	Steady state probability vector of PAP
$\left[{\mathbb{C}}_{mn}\right]$	Subcarrier allocation strategy
$P_{A}$	Transition probability matrix of the PAP
$U^{0}$	Initial utility vector
℧	Set of game theory strategies of the ℝ CRV-SU players and utility vectors’ space
${\mathbb{R}}^{N_{C}}$	Total possible channel assignments
$\left[R_{mn}\right]$	Rate allocation strategy
𝒷	Minimum utility bound
$t_{mn}$	OFDM symbol transmitted by CRV-SU n over the *m*′th subcarrier
$G_{mn}$	Complex circularly-symmetric Gaussian noise
${Ᵽ}_{n}^{max}$	Transmit power constraint to protect potential transmissions of PU
$r_{mn}$	OFDM symbol received at the destination

**Table 2:** List of acronyms.

Acronym	Meaning
BER	Bit error rate
CC	Cognitive cell
CCH	Control channel
CH	Cluster head
CM	Cluster member
CR	Cognitive Radio
CRAVNs	Cognitive Radio-assisted vehicular networks
CR-BS	CR base station
CR-IoVs	Cognitive Radio-enabled IoVs
CRV-SU	Cognitive Radio vehicular secondary user
CS	Cuckoo Search scheme
CSI	Channel state information
DR	Dependent Rounding-based scheme
DSA	Dynamic spectrum access
FCC	Federal Communication Commission
FSA	Fixed spectrum allocation
GPS	Global Positioning System
IEEE	Institute of Electrical and Electronics Engineers
IoVs	Internet of Vehicles
JFI	Jain’s Fairness Index
KKT	Karush–Kuhn–Tucker
LTE	Long-term evolution
MAC	Medium access control
MOCS/D	Multi-objective Optimization based on Decomposition scheme
M-QAM	Multi-level Quadrature Amplitude Modulation
NBS	Nash bargaining solution
OBUs	On-Board Units
Ofcom	UK Office of Communications
OFDMA	Orthogonal frequency division multiple access
PAP	Packet arrival process
PAR	Packet arrival rate
PDP	Power delay profile
PHY	Physical layers
PNE-S	Pure Nash Equilibrium Search scheme
PU	Primary user
QoS	Quality of service
SCHs	Service channels
SINR	Signal-to-interference-and-noise ratio
SNB	Symmetric Nash bargaining
SNBS	Symmetric Nash bargaining solution
SNO-CRAVNET	SNBS OFDMA-based overlay CR-Assisted Vehicular NETwork
TV	Television
UMTS	Universal mobile telecommunication system
V2I	Vehicle-to-roadside infrastructure
V2V	Vehicle-to-vehicle
V2X	Vehicle-to-pedestrian’s handheld devices and others
VANET	Vehicular ad-hoc network
WAVEs	Wireless Access in Vehicular Environments
WG	Working Group
*wrt*	With respect to

**Table 3:** Parameter settings.

Parameter	Setting
SINR threshold, ($\beta_{n}^{min}$)	5 dB
Noise density, ($BN_{0}$)	0.1 dBm
Channel bandwidth, (B)	1.6 GHz
Power threshold, ($P_{n}^{max}$)	1.5 W
PU noise density, $(BN_{0}^{n^{\prime }}$)	0.1 dBm
Distance between *n*′th PU and CR-BS, ($d_{n^{\prime }}^{CR-BS}$)	12 m
Distance between *n*th CRV-SU and the *n*′th PU, ($d_{nn^{\prime }}$)	4 m
PU interference threshold, ($\beta_{PU}^{min}$)	5 dB
Exponent of path attenuation, (υ)	2.5

## Appendix A

### Proofs of Propositions 1, 2, and 3

**Proof of Proposition 1.** In Equation (28), $Ʈ\left({\tilde{C}}_{mn},\;{\tilde{P}}_{mn}\right)$ can be expressed as $\sum_{n=1}^{\mathbb{R}}\ln\left\{\sum_{m=1}^{N_{C}}\left({\tilde{C}}_{mn}\frac{B}{N_{C}}\log_{2}\left\{1+\left({\tilde{P}}_{mn}{\left|a_{mn}\right|}^{2}\varphi /{\tilde{C}}_{mn}\right)\right\}\right)-U_{n}^{0}\right\}$ using Equations (11) and (12). In the above expression, $Ʈ\left({\tilde{C}}_{mn},\;{\tilde{P}}_{mn}\right)$ represents the sum of functions which can be given as $f\left({\tilde{C}}_{mn},\;{\tilde{P}}_{mn}\right)=\ln\left\{\left({\tilde{C}}_{mn}X\log_{2}\left\{1+\left({\tilde{P}}_{mn}Y/\left({\tilde{C}}_{mn}\right)\right)\right\}\right)-Z\right\}$, with X, Y, and Z denoting positive constants. The evaluation of the Hessian matrix $f\left({\tilde{C}}_{mn},\;{\tilde{P}}_{mn}\right)$ shows that $f\left({\tilde{C}}_{mn},\;{\tilde{P}}_{mn}\right)$ is a negative semi-definite. Then, according to [32], by implication, it means that over ${\tilde{C}}_{mn}$ and ${\tilde{P}}_{mn}$, $f\left({\tilde{C}}_{mn},\;{\tilde{P}}_{mn}\right)$ is a concave function. Therefore, it also follows that $Ʈ\left({\tilde{C}}_{mn},\;{\tilde{P}}_{mn}\right)$ is a concave function, given that any non-negative definite combination of concave functions is likewise concave. Additionally, over a convex set, the convex optimization problem expressed in Equations (28)–(34) is determined. Given that each constraint as shown in Equations (29)–(34), according to its affinity determining a convex set, the set defined by each of the constraints is convex, since the intersection of convex sets is convex according to [32]. The Proof of Proposition 1 is completed by the above presented argument. □

**Proof of Proposition 2.** Let the first order derivative of $Ʈ\left({\tilde{C}}_{mn},\;{\tilde{P}}_{mn}\right)$ wrt ${\tilde{C}}_{mn}$ be denoted by ${ƛ}_{mn}\left({\tilde{C}}_{mn}\right)$, where

$${ƛ}_{mn}({\tilde{C}}_{mn})\underset{=}{\mathrm{def}}\frac{\partial \left\{Ʈ\left({\tilde{C}}_{mn},\;{\tilde{P}}_{mn}\right)\right\}}{\partial {\tilde{C}}_{mn}},$$

(A1) $$=\frac{\log_{2}\left(1+\frac{{\tilde{P}}_{mn}{\left|a_{mn}\right|}^{2}\varphi }{{\tilde{C}}_{mn}}\right)-\left\{\frac{{\tilde{P}}_{mn}{\left|a_{mn}\right|}^{2}\varphi }{\ln2\left({\tilde{C}}_{mn}\left({\tilde{P}}_{mn}{\left|a_{mn}\right|}^{2}\varphi \right)\right)}\right\}}{{\tilde{C}}_{mn}\log_{2}\left(1+\frac{{\tilde{P}}_{mn}{\left|a_{mn}\right|}^{2}\varphi }{{\tilde{C}}_{mn}}\right)-\left(\frac{N_{C}U_{n}^{0}}{B}\right)}.$$

With all the ${\tilde{C}}_{mn}$ satisfying $U_{n}^{0}<R_{mn}{\tilde{C}}_{mn}\left({\tilde{C}}_{mn},\;{\tilde{P}}_{mn}\right)$, this leads to $\frac{N_{C}U_{n}^{0}}{B}<{\tilde{C}}_{mn}\log_{2}\left\{1+\frac{{\tilde{P}}_{mn}{\left|a_{mn}\right|}^{2}\varphi }{{\tilde{C}}_{mn}}\right\}$, thereby guaranteeing that the denominator in Equation (A1) is non-negative. Therefore, in Equation (A1), showing that the numerator is likewise non-negative for $0<{\tilde{C}}_{mn}^{min}<{\tilde{C}}_{mn}$ adequately suffices to demonstrate the strictly increasing property, where ${\tilde{C}}_{mn}^{min}$ denotes the unique solution of $\frac{N_{C}U_{n}^{0}}{B}={\tilde{C}}_{mn}\log_{2}\left\{1+\frac{{\tilde{P}}_{mn}{\left|a_{mn}\right|}^{2}\varphi }{{\tilde{C}}_{mn}}\right\}$. Let $y=\frac{{\tilde{P}}_{mn}{\left|a_{mn}\right|}^{2}\varphi }{{\tilde{C}}_{mn}}$, so that, in Equation (A1), the numerator can be expressed using the function $H\left(y\right)=\log_{2}\left\{\left(\left(1+y\right)-y\right)/\ln\left(2\left(1+y\right)\right)\right\}$, where y>0. Hence, when y>0, it can be easily verified that the first order derivative of H(y) over y is non-negative, that is, $\frac{\partial H\left(y\right)}{\partial y}=\frac{y}{\ln\left(2{\left(1+y\right)}^{2}\right)}>0$. Therefore, it follows that H(y) is a strictly increasing function, which can be determined through H(y)>H(0)=0. Therefore, ${ƛ}_{mn}\left({\tilde{C}}_{mn}\right)>0$, and it can be concluded that $Ʈ\left({\tilde{C}}_{mn},\;{\tilde{P}}_{mn}\right)$ strictly increases. Proof of Proposition 2 is completed. □

**Proof of Proposition 3.** The utility function can be expressed with regards to the data-rate vector, that is, $R={\left[R_{1}\left({\tilde{C}}_{m1},\;{\tilde{P}}_{m1}\right),\;R_{2}\left({\tilde{C}}_{m2},\;{\tilde{P}}_{m2}\right),\;\cdots ,\;R_{\mathbb{R}}\left({\tilde{C}}_{m\mathbb{R}},\;{\tilde{P}}_{m\mathbb{R}}\right)\right]}^{H}$ rewritten as $Ʈ\left(R\right)=\prod_{n=1}^{\mathbb{R}}\left\{R_{n}\left({\tilde{C}}_{mn},\;{\tilde{P}}_{mn}\right)-U_{n}^{0}\right\}$, where ${\left[\cdot \right]}^{H}$ represents the vector transpose. In Equation (28), the disagreement points of $Ʈ\left({\tilde{C}}_{mn},\;{\tilde{P}}_{mn}\right)$, for equilibrium analysis, are set to $U_{n}^{0}=0$. Therefore, Ʈ(R) is minimized to $Ʈ\left(R\right)=\prod_{n=1}^{\mathbb{R}}R_{n}\left({\tilde{C}}_{mn},\;{\tilde{P}}_{mn}\right)$. The objective function with regards to Proposition 2 can be expressed as ln{Ʈ(R)}. Therefore, if ${\tilde{C}}_{mn}$, $R={\left[R_{1}\left({\tilde{C}}_{m1},\;{\tilde{P}}_{m1}\right),\;R_{2}\left({\tilde{C}}_{m2},\;{\tilde{P}}_{m2}\right),\;\cdots ,\;R_{\mathbb{R}}\left({\tilde{C}}_{m\mathbb{R}},\;{\tilde{P}}_{m\mathbb{R}}\right)\right]}^{H},$ and ${\tilde{P}}_{mn}$ are the optimal channel, achievable data-rate, and transmit power allocation, respectively, then it follows that, at $R=R^{*}$, the following condition stands:

(A2) $$\overset{\mathbb{R}}{\underset{n=1}{\sum }}\frac{\partial \ln\left\{Ʈ\left(R\right)\right\}}{\partial R_{n}}{|}_{R_{n}-R_{n}^{*}}\left(R_{n}-R_{n}^{*}\right)=\overset{\mathbb{R}}{\underset{n=1}{\sum }}\frac{R_{n}-R_{n}^{*}}{R_{n}^{*}}\leq 0.$$

In Equation (A2), the condition ensures proportional fairness [34], since the objective function cannot be improved by each movement in the line of $R-R^{*}$ at the optimal data-rate vector $R^{*}$. Therefore, proportionally fair resource allocation is guaranteed by the optimal solution. Proof of Proposition 3 is completed. □

(A3) $$\begin{array}{ll} \tilde{𝓁}({\tilde{C}}_{mn},\;{\tilde{P}}_{mn},\;𝓀,{₼}_{n},{𝒻}_{n},\;{𝒷}_{n}) & \\ & =\{\overset{\mathbb{R}}{\underset{n=1}{\sum }}\ln\{\overset{N_{C}}{\underset{m=1}{\sum }}({\tilde{C}}_{mn}\frac{B}{N_{C}}\log_{2}(1+\frac{{\tilde{P}}_{mn}{\left|a_{mn}\right|}^{2}\varphi }{{\tilde{C}}_{mn}}))-U_{n}^{0}\}\\ & -𝓀(\overset{\mathbb{R}}{\underset{n=1}{\sum }}\overset{N_{C}}{\underset{m=1}{\sum }}{\tilde{P}}_{mn}-P_{Tot})-\overset{N_{C}}{\underset{m=1}{\sum }}{₼}_{n}(\overset{\mathbb{R}}{\underset{n=1}{\sum }}{\tilde{C}}_{mn}-1)\\ & +{𝒻}_{n}(\overset{N_{C}}{\underset{m=1}{\sum }}{\tilde{P}}_{mn}{\left|a_{mn}\right|}^{2}-{Ᵽ}_{n}^{min})+{𝒷}_{n}({Ᵽ}_{n}^{max}-\overset{N_{C}}{\underset{m=1}{\sum }}{\tilde{P}}_{mn})\}. \end{array}$$

## Appendix B

### Proofs of Theorems 2, 3, 4, and 5

The convex optimization problem’s Lagrangian function $\tilde{𝓁}$ as expressed in Equations (28)–(34) is defined as shown in Equation (A3) above, where 𝓀≥0, ${₼}_{n}\geq 0$, ${𝒻}_{n}\geq 0$, and ${𝒷}_{n}\geq 0$ denote the four constraints’ (i.e., Equation (30) and (32)–(34)) Lagrangian multipliers, respectively. By applying the Karush–Kuhn–Tucker (KKT) conditions according to [35], the optimal subcarrier allocation index ${\tilde{\mathbb{C}}}_{mn}^{*}$ is obtained through the differentiation of $\tilde{𝓁}$ over ${\tilde{\mathbb{C}}}_{mn}$ in Equation (A3) above, so that,

$$\frac{\partial \tilde{𝓁}}{\partial {\tilde{\mathbb{C}}}_{mn}\;}{|}_{\left({\tilde{\mathbb{C}}}_{mn},{\tilde{P}}_{mn},{₼}_{n},𝓀,{𝒻}_{n},\;{𝒷}_{n}\right)=\left({\tilde{\mathbb{C}}}_{mn}^{*},{\tilde{P}}_{mn}^{*},\;{₼}_{n}^{*},{𝓀}^{*},\;{𝒻}_{n}^{*},\;{𝒷}_{n}^{*}\right)}=0\Rightarrow {ƛ}_{mn}\left({\tilde{\mathbb{C}}}_{mn}^{*}\right)={₼}_{m}^{*},\;\forall m,n$$

Through the differentiation of ${ƛ}_{mn}\left({\tilde{\mathbb{C}}}_{mn}^{*}\right)$ wrt ${\tilde{\mathbb{C}}}_{mn}^{*}$, it is shown that $\partial {ƛ}_{mn}\left({\tilde{\mathbb{C}}}_{mn}^{*}\right)/\partial {\tilde{\mathbb{C}}}_{mn}^{*}<0$, ∀ ${\tilde{\mathbb{C}}}_{mn}^{min}<{\tilde{\mathbb{C}}}_{mn}^{*}$. This further shows that ${ƛ}_{mn}\left({\tilde{\mathbb{C}}}_{mn}^{*}\right)$ is strictly non-increasing, while its inverse function, that is, ${ƛ}_{mn}^{-1}\left({\tilde{\mathbb{C}}}_{mn}^{*}\right)$, exists for ${\tilde{\mathbb{C}}}_{mn}^{*}>0$. Furthermore, by differentiating Equation (A3) wrt ${₼}_{m}^{*}$ yields $\sum_{n=1}^{\mathbb{R}}{\tilde{\mathbb{C}}}_{mn}^{*}-1=0$. Note that ${ƛ}_{mn}^{-1}\left({₼}_{m}^{*}\right)={\tilde{\mathbb{C}}}_{mn}^{*}$ and $\sum_{n=1}^{\mathbb{R}}{\tilde{\mathbb{C}}}_{mn}^{*}=1$ result in $\sum_{n=1}^{\mathbb{R}}{ƛ}_{mn}^{-1}\left({₼}_{m}^{*}\right)=1$, ∀n.

**Proposition A1.***Let*$ɸ\left({₼}_{m}^{*}\right)=\sum_{n=1}^{\mathbb{R}}{ƛ}_{mn}^{-1}\left({₼}_{m}^{*}\right)$*. Then, there exists an inverse function for*$ɸ\left({₼}_{m}^{*}\right)$.

**Proof.** Recall that $\partial {ƛ}_{\mathrm{mn}}\left({\tilde{\mathbb{C}}}_{\mathrm{mn}}^{*}\right)/\partial {\tilde{\mathbb{C}}}_{\mathrm{mn}}^{*}<0$. Then, it follows that

$$\frac{\partial ɸ\left({₼}_{m}^{*}\right)}{\partial {₼}_{m}^{*}}=\overset{\mathbb{R}}{\underset{n=1}{\sum }}\frac{\partial {ƛ}_{mn}^{-1}\left({₼}_{m}^{*}\right)}{\partial {₼}_{m}^{*}}=\overset{\mathbb{R}}{\underset{n=1}{\sum }}\left\{\frac{1}{\left(\frac{\partial {ƛ}_{mn}\left({\tilde{\mathbb{C}}}_{mn}^{*}\right)}{\partial {\tilde{\mathbb{C}}}_{mn}^{*}}\right)}\right\}<0,\;\forall n.$$

Therefore, $ɸ\left({₼}_{m}^{*}\right)$ is an entirely strictly non-increasing function. Proof of Proposition 4 is completed. The optimum Lagrangian multiplier, ${₼}_{m}^{*}$, is obtained from Proposition 4, which is given in Equation (A4) below:

(A4) $${₼}_{m}^{*}={ɸ}^{-1}\left(1\right),\;\forall n.$$

Therefore, the globally optimum solution of ${\tilde{\mathbb{C}}}_{mn}^{*}$ is obtained from Equation (A4) via the expression

(A5) $${\tilde{\mathbb{C}}}_{mn}^{*}={ƛ}_{mn}^{-1}\left({₼}_{m}^{*}\right),\;\forall m,\;n.$$

The Proof of Theorem 2 is completed. □

Applying the KKT conditions and differentiating $\tilde{𝓁}$ wrt ${\tilde{P}}_{mn}$ in Equation (A3) yields ${\tilde{P}}_{mn}^{*}$ (i.e., the optimal transmit power allocation index). Therefore,

(A6) $$\begin{array}{ll} \frac{\partial \tilde{𝓁}}{\partial {\tilde{P}}_{mn}\;} & {|}_{\left({\tilde{\mathbb{C}}}_{mn},{\tilde{P}}_{mn},{₼}_{n},𝓀,{𝒻}_{n},\;{𝒷}_{n}\right)=\left({\tilde{\mathbb{C}}}_{mn}^{*},{\tilde{P}}_{mn}^{*},\;{₼}_{n}^{*},{𝓀}^{*},\;{𝒻}_{n}^{*},\;{𝒷}_{n}^{*}\right)}=0\\ & \Rightarrow \left(1+\frac{{\tilde{P}}_{mn}^{*}{\left|a_{mn}\right|}^{2}\varphi }{{\tilde{\mathbb{C}}}_{mn}^{*}}\right)\left\{\left({\tilde{\mathbb{C}}}_{mn}^{*}\frac{B}{N_{C}}\log_{2}\left(1+\frac{{\tilde{P}}_{mn}^{*}{\left|a_{mn}\right|}^{2}\varphi }{{\tilde{\mathbb{C}}}_{mn}^{*}}\right)\right)-U_{n}^{0}\right\}\\ & =\frac{B{\left|a_{mn}\right|}^{2}\varphi }{N_{C}\ln2\left({𝓀}^{*}-{𝒻}_{n}^{*}{\left|a_{mn}\right|}^{2}+{𝒷}_{n}^{*}\right)} \end{array}$$

Equation (A6) is noted as a transcendental algebraic equation *wrt* the optimal transmit power allocation index ${\tilde{P}}_{mn}^{*}$. This type of equation is usually solved by related studies through recursive numerical methods. However, recursive numerical methods are known to be computationally intensive [36,37]. Hence, an analytical solution of ${\tilde{P}}_{mn}^{*}$ is provided in Equation (A6), as shown below. Let

(A7) $${ᶀ}_{mn}=1+\frac{{\tilde{P}}_{mn}^{*}{\left|a_{mn}\right|}^{2}\varphi }{{\tilde{\mathbb{C}}}_{mn}^{*}},$$

(A8) $$X_{mn}=\frac{B{\left|a_{mn}\right|}^{2}\varphi }{N_{C}\ln2\left({𝓀}^{*}-{𝒻}_{n}^{*}{\left|a_{mn}\right|}^{2}+{𝒷}_{n}^{*}\right)},$$

and

(A9) $${Þ}_{mn}=2^{\left\{\frac{U_{n}^{0}}{{\tilde{\mathbb{C}}}_{mn}^{*}\left(B/N_{C}\right)}\right\}},\;{Þ}_{mn}\geq 1.$$

Therefore, Equation (A6) can be further simplified as shown below:

(A10) $${\tilde{\mathbb{C}}}_{mn}^{*}\frac{B}{N_{C}}\log_{2}{\left(\frac{{ᶀ}_{mn}}{{Þ}_{mn}}\right)}^{{ᶀ}_{mn}}=X_{mn}.$$

**Proof.** Both sides of Equation (A10) multiplied by $\frac{1}{{Þ}_{mn}}$ yields

(A11) $${\tilde{\mathbb{C}}}_{mn}^{*}\frac{B}{N_{C}}\log_{2}{\left(\frac{{ᶀ}_{mn}}{{Þ}_{mn}}\right)}^{\left(\frac{{ᶀ}_{mn}}{{Þ}_{mn}}\right)}=\frac{X_{mn}}{{Þ}_{mn}}.$$

Now, let $ᵹ=\frac{{ᶀ}_{mn}}{{Þ}_{mn}}$. Then, Equation (A11) becomes

(A12) $${ᵹ}^{ᵹ}=2^{\left\{\frac{N_{C}X_{mn}}{{\tilde{\mathbb{C}}}_{mn}^{*}\left(B{Þ}_{mn}\right)}\right\}}.$$

By applying the properties of the Lambert-Ⱳ function [38,39], Equation (A12) yields

(A13) $$ᵹ=e^{\left\{Ⱳ\left(\ln\left(2^{\left(\frac{N_{C}X_{mn}}{{\tilde{\mathbb{C}}}_{mn}^{*}\left(B{Þ}_{mn}\right)}\right)}\right)\right)\right\}},$$

where Ⱳ(·) represents the Lambert-Ⱳ function. Then, substituting ᵹ with $\frac{{ᶀ}_{mn}}{{Þ}_{mn}}$ in Equation (A13) gives

(A14) $${ᶀ}_{mn}={Þ}_{mn}\cdot e^{\left\{Ⱳ\left(\ln\left(2^{\left(\frac{N_{C}X_{mn}}{{\tilde{\mathbb{C}}}_{mn}^{*}\left(B{Þ}_{mn}\right)}\right)}\right)\right)\right\}}.$$

Therefore, by relating Equations (A7) and (A14), the optimal transmit power allocation ${\tilde{P}}_{mn}^{*}$ of the proposed novel SNO-CRAVNET is determined to in Equation (36). Proof of Theorem 3 is completed. □

**Proof.** The optimal transmit power allocation ${\tilde{P}}_{mn}^{*}$ as contained in Equation (36) signifies the exact measure of the optimal transmit power on subcarrier m of all the CRV-SU n. Therefore, if ${\tilde{P}}_{mn^{\prime }}^{*}<{\tilde{P}}_{mn}^{*}$, then the CRV-SU n requires more transmit power ${\tilde{P}}_{mn}^{*}$ compared to CRV-SU $n^{\prime }$, so as to be able to transmit the same amount of message on subcarrier n. Hence, the CRV-SU that will be selected is the one that requires the minimum transmit power. To achieve this, a linear search is performed amongst the dynamically available $N_{C}$ subcarriers of the optimum CRV-SU $n^{*}$, which is given by $n^{*}=\arg\;\min{\tilde{P}}_{mn}^{*}$. Proof of Theorem 4 is completed. □

**Proof.** Furthermore, based on Equation (37), the binary representation of Equation (35) gives the proposed novel SNO-CRAVNET optimal subcarrier scheduling strategy ${\mathbb{C}}_{N_{C}\times \mathbb{R}}^{*}\left[O_{N_{C}\times \mathbb{R}}\right]=\left[{\mathbb{C}}_{mn}^{*}\right]$; for instance, ${\mathbb{C}}_{mn}^{*}=0$ when $n\neq n^{*}$, and ${\mathbb{C}}_{mn}^{*}=1$ when $n=n^{*}$. Proof of Theorem 5 is completed. □
